# Activation and Purification of *ß*‐Glucocerebrosidase by Exploiting its Transporter LIMP‐2 – Implications for Novel Treatment Strategies in Gaucher's and Parkinson's Disease

**DOI:** 10.1002/advs.202401641

**Published:** 2024-04-26

**Authors:** Jan Philipp Dobert, Simon Bub, Rebecca Mächtel, Dovile Januliene, Lisa Steger, Martin Regensburger, Sibylle Wilfling, Jia‐Xuan Chen, Mario Dejung, Sonja Plötz, Ute Hehr, Arne Moeller, Philipp Arnold, Friederike Zunke

**Affiliations:** ^1^ Department of Molecular Neurology University Hospital Erlangen Friedrich‐Alexander‐University Erlangen‐Nürnberg (FAU) 91054 Erlangen Germany; ^2^ Department of Structural Biology Osnabrueck University 49076 Osnabrueck Germany; ^3^ Deutsches Zentrum Immuntherapie (DZI) Friedrich‐Alexander‐Universität Erlangen‐Nürnberg (FAU) 91054 Erlangen Germany; ^4^ Center for Human Genetics Regensburg 93059 Regensburg Germany; ^5^ Institute of Molecular Biology (IMB) 55128 Mainz Germany; ^6^ Institute of Anatomy Functional and Clinical Anatomy Friedrich‐Alexander‐University Erlangen‐Nürnberg (FAU) 91054 Erlangen Germany

**Keywords:** β‐glucocerebrosidase, Gaucher's disease (GD), GCase, LIMP‐2, lysosomes, Parkinson's disease (PD), protein purification

## Abstract

Genetic variants of *GBA1* can cause the lysosomal storage disorder Gaucher disease and are among the highest genetic risk factors for Parkinson's disease (PD). *GBA1* encodes the lysosomal enzyme beta‐glucocerebrosidase (GCase), which orchestrates the degradation of glucosylceramide (GluCer) in the lysosome. Recent studies have shown that GluCer accelerates *α*‐synuclein aggregation, exposing GCase deficiency as a major risk factor in PD pathology and as a promising target for treatment. This study investigates the interaction of GCase and three disease‐associated variants (p.E326K, p.N370S, p.L444P) with their transporter, the lysosomal integral membrane protein 2 (LIMP‐2). Overexpression of LIMP‐2 in HEK 293T cells boosts lysosomal abundance of wt, E326K, and N370S GCase and increases/rescues enzymatic activity of the wt and E326K variant. Using a novel purification approach, co‐purification of untagged wt, E326K, and N370S GCase in complex with His‐tagged LIMP‐2 from cell supernatant of HEK 293F cells is achieved, confirming functional binding and trafficking for these variants. Furthermore, a single helix in the LIMP‐2 ectodomain is exploited to design a lysosome‐targeted peptide that enhances lysosomal GCase activity in PD patient‐derived and control fibroblasts. These findings reveal LIMP‐2 as an allosteric activator of GCase, suggesting a possible therapeutic potential of targeting this interaction.

## Graphical abstract

This study explores the interaction of the lysosomal *β*‐glucocerebrosidase with its transporter LIMP‐2. LIMP‐2 overexpression boosts lysosomal transport and activity of wild type *β*‐glucocerebrosidase and the Parkinson's disease‐associated E326K variant. This interaction is then exploited to purify *β*‐glucocerebrosidase in complex with LIMP‐2 and to design a lysosome‐targeted, LIMP‐2‐derived peptide that increases lysosomal *β*‐glucocerebrosidase activity in control and patient‐derived fibroblasts.

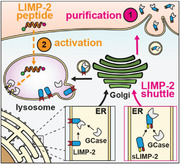

## 1 Introduction

Beta‐glucocerebrosidase (GCase) is a lysosomal enzyme involved in the degradation of the sphingolipid glucosylceramide (GluCer).^[^ ^1^, ^2^ ^]^ Impairment of the enzyme's lysosomal activity due to mutations in the *GBA1* gene is the monogenic cause of the most common lysosomal storage disorder, Gaucher's disease (GD).^[^ ^3^ ^]^ In addition, GCase malfunction has recently emerged as a key pathological mechanism in the neurodegenerative disorder Parkinson's disease (PD).^[^ ^2^ ^]^ In the context of both diseases, rescuing lysosomal GCase activity by discovering GCase‐activating compounds/mechanisms has become a principal target of pharmacological research.^[^ ^4^, ^5^, ^6^, ^7^, ^8^, ^9^, ^10^, ^11^, ^12^, ^13^ ^]^

The link between GCase and PD was first established when studies found an increased prevalence of PD in GD patients and their relatives.^[^ ^14^, ^15^, ^16^ ^]^ Carriers of *GBA1* mutations exhibit an up to 20‐fold increased risk of developing PD, making *GBA1* one of the highest genetic risk factors.^[^ ^17^, ^18^ ^]^ The most common PD‐associated GCase variants are p.N370S and p.L444P,^[^ ^3^, ^19^ ^]^ which are also prevalent in GD, and p.E326K, a variant that does not appear to cause GD.^[^ ^20^ ^]^ All three variants are characterized by compromised stability and/or enzyme activity. The L444P variant shows impaired stability and highly impaired activity, N370S is stable with low residual activity and E326K is stable while retaining ≈40% residual activity.^[^ ^21^, ^22^, ^23^ ^]^ This residual activity of the E326K variants appears to be sufficient to prevent GD. GCase and PD pathology converge by the effect of GluCer on the synaptic protein *α*‐synuclein,^[^ ^24^ ^]^ which has been shown to aggregate into cell toxic conformers in PD and is accompanied by neuronal cell death.^[^ ^4^, ^25^, ^26^ ^]^ GluCer and *α*‐synuclein form a bidirectional pathogenic loop, where GluCer promotes *α*‐synuclein aggregation, which in turn interferes with cellular trafficking, decreasing lysosomal function and thus GCase activity.^[^ ^5^, ^27^, ^28^ ^]^ Hence, *GBA1* mutations increase PD risk by accelerating *α*‐synuclein aggregation as also shown in mice.^[^ ^29^, ^30^ ^]^ Additional pathological gain‐of‐function mechanisms have been described for certain *GBA1* variants, such as ER stress,^[^ ^31^ ^]^ blockage of chaperone‐mediated autophagy^[^ ^32^ ^]^ or lipid droplet accumulation.^[^ ^33^ ^]^ It is worth mentioning that *GBA1* null mutations as well as GCase inhibitory studies also recapitulate *α*‐synuclein aggregation and PD pathology,^[^ ^27^, ^34^ ^]^ indicating a complex interplay of *GBA1*‐induced loss‐ and gain‐of‐function mechanisms.

To maintain GCase function, delivery of the enzyme to the lysosome is crucial. Unlike almost all other lysosomal enzymes, GCase harbors no lysosomal targeting signal in the form of a mannose‐6‐phosphate (M6P) residue.^[^ ^35^, ^36^ ^]^ GCase is one of the few lysosomal hydrolases that is M6P‐independent and instead solely relies on the lysosomal integral membrane protein type 2 (LIMP‐2) to reach the lysosome.^[^ ^35^, ^37^, ^38^, ^39^ ^]^ Data indicates the interaction of both proteins within the endoplasmic reticulum (ER) to form a transporting complex, further undergoing post‐translational modification along the secretory pathway (e.g., glycosylation) before being directed to the endosomal compartments via a lysosomal signaling sequence on LIMP‐2.^[^ ^40^ ^]^ In a LIMP‐2‐deficient mouse model, lysosomal GCase levels are heavily reduced, confirming the importance of LIMP‐2 for GCase transport and function. Interestingly, this LIMP‐2‐deficient mouse model also exhibits a‐syn pathology, further underlining the mechanistic interplay of LIMP‐2, GCase, and *α*‐synuclein.^[^ ^41^ ^]^ In a previous study, we identified an additional function of LIMP‐2 with regard to GCase, as we could identify enhanced protein stability and GCase activity after the formation of the LIMP‐2/GCase complex.^[^ ^42^ ^]^ Despite the importance of the LIMP‐2‐GCase protein complex, details about the molecular mechanisms of this protein–protein interaction remain unclear. To date, although single protein structures have been solved for both proteins,^[^ ^39^, ^43^ ^]^ no molecular resolution of the complex has been obtained.

In this study, we investigate the interaction of wild type (wt) GCase as well as its disease‐associated variants E326K, N370S, and L444P with their lysosomal transporter LIMP‐2 in order to unravel the role of complex formation in the context of disease. To accomplish this, we performed overexpression studies of GCase variants alongside LIMP‐2 in a HEK 293T cell model and characterized primary human fibroblast cell lines from PD and GD patients, focusing on lysosomal delivery and activity of GCase. Interestingly, only the PD‐associated GCase‐E326K variant was susceptible to rescue via LIMP‐2, as both its lysosomal localization and activity could be enhanced.

Further, we engineered a soluble, secreted LIMP‐2 construct to enable purification of the LIMP‐2/GCase protein complex from the supernatant of HEK 293F cells, also allowing for the co‐purification of untagged/unmodified GCase protein. As this purification protocol provides the physiological formation of the complex, it permits screening of GCase modifiers that will not interfere with the lysosomal transport of the enzyme. Further, we consider the purification of GCase variants in complex with LIMP‐2 beneficial for stability‐ and/or maturation‐impaired GCase variants.^[^ ^21^ ^]^

Based on our recent data,^[^ ^42^ ^]^ we optimized a small, lysosome‐targeted peptide that mimics the binding of LIMP‐2 to GCase. Application of the LIMP‐2‐derived peptide increased activity of wt and PD‐associated E326K GCase in vitro, as well as in lysosomes of PD patient fibroblasts harboring *GBA1*‐E326K mutations. This study reveals LIMP‐2 and the LIMP‐2‐derived peptide as an allosteric activator of GCase, potentially opening up new approaches for the development of urgently needed novel therapeutic strategies in order to rescue GCase activity in GD and PD.

## 2 Results

### 2.1 LIMP‐2 Increases Intracellular Transport and Lysosomal Activity of Select GCase Variants

**Figure 1:**
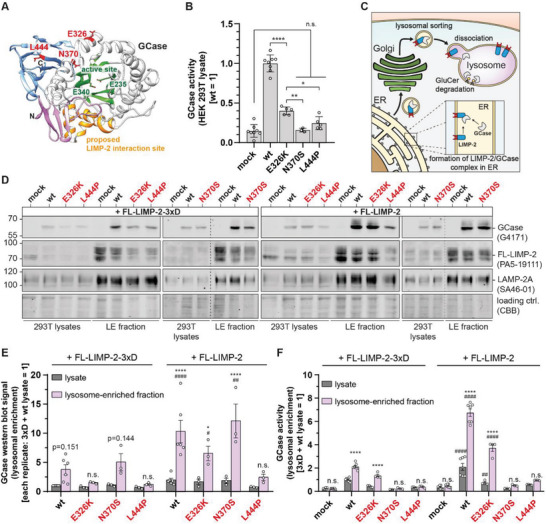
Interaction of GCase variants with their lysosomal transporter LIMP‐2. A) Crystal structure of GCase (PDB: 5LVX).^[^ ^44^ ^]^ The domains and motifs are colored as follows: pink: antiparallel beta‐sheet (domain 1); grey: TIM barrel (domain 2) containing active site (green); blue: beta‐barrel (domain 3);^[^ ^45^ ^]^ orange: proposed LIMP‐2‐binding motif.^[^ ^42^ ^]^ Single amino acids comprising the active site, as well as amino acids exchanged in common disease‐associated GCase variants E326K, N370S, and L444P are labeled and highlighted. B) GCase activity in cell lysates of HEK 293T cells overexpressing GCase variants (n  =  3‐8; mock: 8; wt: 8; E326K: 5; N370S: 3; L444P: 5; individual transfections). All disease‐associated variants show significantly reduced activity. E326K shows a residual activity of 40.0 ± 2.2%, N370S, and L444P do not differ significantly from the mock. C) Illustration of the lysosomal transport of GCase and its interaction with LIMP‐2. GCase binds to LIMP‐2 in the ER to form a transporting complex. After trafficking through the Golgi, the LIMP‐2/GCase complex is sorted to lysosomal compartments where low pH triggers complex dissociation. D) Western blot analyses of whole‐cell lysates and LE fractions of HEK 293T cells expressing GCase variants and FL‐LIMP‐2 or FL‐LIMP‐2‐3xD as a control. LE fractions are higher in GCase, LIMP‐2, and the lysosomal protein LAMP‐2A. Enriched fractions of cells overexpressing FL‐LIMP‐2 show increased levels of GCase. Blots containing a dashed line were spliced for a more comprehensive data presentation (both parts are from the same image). E) Quantification of western blot signal from lysate and LE HEK 293T samples (Figure 1D) (n  =  3‐6; mock: 6; wt: 6; E326K: 3; N370S: 3; L444P: 3; individual cell harvests). The abundance of wt, E326K, and N370S GCase was significantly increased in LE fractions when FL‐LIMP‐2 was co‐expressed compared to the co‐expression of the non‐binding 3xD control. In general, the GCase signal was increased in LE fractions but did not reach statistical significance for FL‐LIMP‐2‐3xD samples and FL‐LIMP‐2 + L444P. F) GCase activity in cell lysates and LE fractions of HEK 293T cells overexpressing GCase variants and FL‐LIMP‐2 or FL‐LIMP‐2‐3xD (n  =  3‐7; mock: 7; wt: 7; E326K: 4; N370S: 3; L444P: 4; individual cell harvests). Overexpression of FL‐LIMP‐2 resulted in an increase of GCase activity in the lysate itself, but also in the LE fraction for wt GCase and the E326K variant, while no significant differences were observed for the N370S and L444P variant. Asterisks (*) indicate significant differences between cell lysate and enriched fractions. Pound signs (#) indicate significant differences in relation to the respective 3xD control sample (example: wt + FL‐LIMP‐2 lysate versus wt + FL‐LIMP‐2‐3xD lysate). Statistics: replicates (dots) with a mean (column) ± SEM (B,E,F). Tests: One‐way ANOVA with Tukey's multiple comparison test (B); Two‐way ANOVA with Tukey's multiple comparison test (E,F). * p < 0.05, ** p < 0.01, *** p < 0.001; **** p < 0.0001; ## p < 0.01, #### p < 0.0001, n.s.: not significant.

The disease‐associated GCase variants E326K, N370S, and L444P are all characterized by an impaired enzymatic activity,^[^ ^21^ ^]^ although the amino acid exchanges are not located within the active site or the proposed interaction interface with LIMP‐2^[^ ^42^ ^]^ (**Figure** **1**A). After Overexpression of these GCase variants in HEK293F cells, all were detectable in cell lysates by western blot (Figure S1A, Supporting Information). However, activity was strongly impaired for all GD‐ and PD‐associated variants when compared to wt, with activities of N370S and L444P GCase being not significantly different to mock‐transfected control cells (Figure 1B). Restoring GCase enzymatic activity may constitute an effective approach to rescue lysosomal function in GCase‐associated pathologies like GD and PD.

LIMP‐2 targets GCase to the lysosome (Figure 1C)^[^ ^35^ ^]^ and has been shown to directly increase GCase activity in vitro.^[^ ^42^ ^]^ This suggests an additional function of LIMP‐2 as a chaperone and/or allosteric activator of GCase in addition to its transporter function. Using commercially available recombinant proteins, we confirmed the interaction of LIMP‐2 and GCase via multiscale thermophoresis (MST) (Figure S1B, Supporting Information) as well as a concentration‐dependent effect of LIMP‐2 on GCase activity using a 4‐MU‐based activity assay (Figure S1C, Supporting Information). Validating the positive effect of LIMP‐2 on GCase function in vitro, we investigated whether an increased level of LIMP‐2 would also benefit intracellular GCase localization and activity. For this, GCase wt, E326K, N370S, and L444P were transiently expressed in HEK 293T cells in a co‐transfection approach together with a full‐length, membrane‐anchored wt LIMP‐2 (further referred to as FL‐LIMP‐2) or with a non‐GCase‐binding FL‐LIMP‐2 variant (FL‐LIMP‐2‐3xD) as a negative control. The LIMP‐2‐3xD variant contains three amino acid exchanges in helix 5 (part of the proposed GCase‐LIMP‐2 interface; Figure S1D,E, Supporting Information), thereby abolishing binding to GCase.^[^ ^42^ ^]^ After co‐expression, we enriched the lysosomal fraction from cell homogenates to investigate the intracellular transport of GCase and LIMP‐2. For this, we compared whole‐cell lysates and lysosome‐enriched (LE) fractions of FL‐LIMP‐2 and FL‐LIMP‐2‐3xD samples utilizing western blot and GCase‐activity analyses. In comparison to whole‐cell lysates, LE fractions exhibited increased and constant levels of the lysosomal marker lysosome‐associated membrane protein 2 variant A (LAMP‐2A), suggesting successful enrichment of lysosomes (Figure 1D). Upon co‐expression of FL‐LIMP‐2, lysosomal levels of wt, E326K, and N370S GCase exceeded those of the FL‐LIMP‐2‐3xD control, indicating an increased transport of these variants to the lysosome (Figure 1D,E). Importantly, enzyme activity in LE fractions showed significant increases for GCase wt and the PD‐associated E326K variant when co‐expressed with FL‐LIMP‐2 (Figure 1F). Although an increased lysosomal transport was detected for the N370S variant, no increase in enzymatic activity could be determined. For the GCase L444P variant, both transport and activity could not be rescued by LIMP‐2, suggesting a severe folding defect probably already within the ER. Notably, expression levels of FL‐LIMP‐2 and FL‐LIMP‐2‐3xD (Figure S1F, Supporting Information) were decreased when co‐expressed with L444P, suggesting either co‐degradation or a negative effect of the L444P variant on the ER.

Taken together, we validated the activating effect of LIMP‐2 on lysosomal GCase enzyme activity in vitro and in a cellular setup in which we also show the importance of LIMP‐2 as a lysosomal transporter for GCase. We observed increased lysosomal abundance and activity of the PD‐associated GCase E326K variant after co‐expression with LIMP‐2, indicating that the E326K variant is susceptible to rescue via LIMP‐2.

### 2.2 Primary Human Fibroblasts Harboring GBA1 Variants Show Altered GCase Levels, Activity and Co‐Localization with LIMP‐2

**Figure 2:**
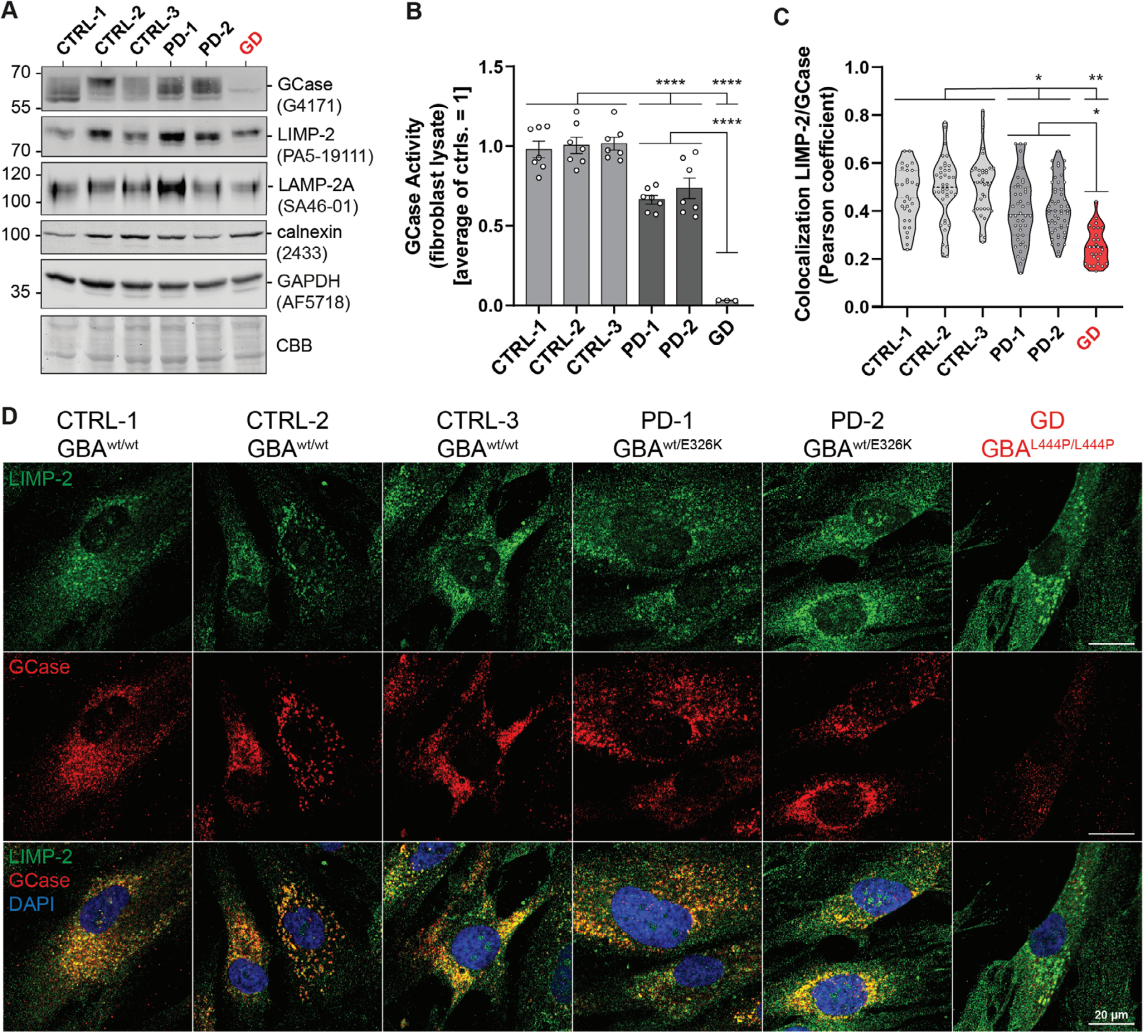
Characterization of primary human fibroblasts of controls and patients with PD and GD. A) Western blot of whole cell lysates from control (CTRL‐1,2,3), PD patient‐derived (PD‐1,2), and GD patient‐derived (GD) primary human fibroblasts. Quantitative analysis of signals can be found in Figure S3 (Supporting Information). GCase levels were comparable between control and PD but diminished in GD (Figure S3A, Supporting Information). LIMP‐2 levels were increased in PD compared to controls (Figure S3B, Supporting Information). LAMP‐2A and calnexin levels varied between cell lines and did not show any significant trend between groups (Figure S3C,D, Supporting Information). GAPDH and CBB staining of the gel are presented as loading controls. B) GCase activity in whole‐cell lysates of control, PD, and GD fibroblast cell lines (n  =  3‐7; CTRL‐1,2,3: 7; PD‐1,2: 7; GD: 3; individual cell harvests). The E326K lines PD‐1 and PD‐2 showed significantly lower GCase activity (66.41±2.69% and 73.56±6.50%) compared to the control lines. In contrast, activity in the GD line (GBA1^L444P/L444P^) was almost fully abolished (3.11±0.11% residual activity). C) Colocalization of LIMP‐2 and GCase in primary human fibroblasts determined via Pearson's correlation coefficient (n  =  CTRL‐1: 30; CTRL‐2: 38; CTRL‐3: 33; PD‐1: 44; PD‐2: 51; GD: 27; individual cells from multiple images). PD patient fibroblasts PD‐1 and PD‐2 show mildly reduced colocalization of LIMP‐2 and GCase compared to control lines. In the GD patient line, colocalization was diminished even further. D) Representative immunofluorescence images of primary human fibroblast lines. Objective magnification: 40x. Green: LIMP‐2; red: GCase, blue: DAPI. All lines show the vesicular distribution of LIMP‐2, indicating lysosomes. Control and PD lines show visible colocalization of GCase with the LIMP‐2 signal. In GD fibroblasts, the GCase signal was less intense and less granular, representing lower expression and lysosomal localization. Statistics: replicates (dots) with mean (column) ± SEM (B); violin plot with median (dashed line) and quartiles (dotted line) (C). Tests: Nested one‐way ANOVA with Tukey's multiple comparison test (B,C). * *p* < 0.05; ** *p* < 0.01; **** *p* < 0.0001.

To investigate the interaction of GCase and LIMP‐2 in a more disease‐relevant context, we utilized six human primary fibroblast cell lines. Three controls (CTRL‐1, CTRL‐2, CTRL‐3) and two PD patient‐derived lines (PD‐1, PD‐2) were obtained at our outpatient clinic. Exome sequencing confirmed *GBA1^wt/wt^* for all controls and *GBA1^wt/E326K^* for both PD lines (Figure S2, Supporting Information). In addition, we obtained a fibroblast line of a GD patient (*GBA1^L444P/L444P^*) from the Coriell Institute for Medical Research as a reference line for highly pathological GCase dysfunction. Both PD lines showed similar GCase levels in western blot analysis compared to the three CTRL lines (**Figure** **2**A; Figure S3A, Supporting Information). However, GCase activity in whole‐cell lysates of PD lines was diminished (66.41 ± 2.69% and 73.56 ± 6.50% residual activity compared to controls, Figure 2B). In contrast, the GD line showed strongly reduced GCase protein levels and only 3.11 ± 0.11% residual lysate activity compared to controls. Interestingly, western blot analysis revealed elevated levels of LIMP‐2 in PD lines compared to controls (Figure 2A; Figure S3B, Supporting Information). LAMP‐2A levels as a general lysosomal marker showed high variance between lines and did not differ significantly between groups (Figure S3C, Supporting Information). As impairments of proper ER function are associated with PD^[^ ^28^ ^]^ and GCase variants have been shown to cause ER stress,^[^ ^8^, ^31^ ^]^ we analyzed the calcium‐dependent molecular chaperone calnexin in our cell lines. Calnexin is important for maintaining protein folding and quality control, particularly of N‐linked glycosylated proteins, including GCase.^[^ ^46^, ^47^ ^]^ Calnexin levels varied between lines and we observed no significant difference between groups (Figure S3D, Supporting Information). Nonetheless, the GD line (GBA1^L444P/L444P^) exhibited the highest levels of calnexin, possibly indicating ER stress as previously published for this variant.^[^ ^48^ ^]^

In order to visualize alterations in the interaction of GCase and LIMP‐2 in these lines, we performed immunofluorescence and calculated co‐localization of GCase and LIMP‐2 in all cell lines via Pearson's correlation (Figure 2C,D). All lines showed a positive correlation coefficient *r* for GCase and LIMP‐2 signal. Co‐localization of GCase and LIMP‐2 was mildly reduced in the PD lines and strongly reduced in the GD line when compared to the controls. The intensity of GCase staining was also highly diminished in the GD line (Figure 2D), corresponding with the decreased protein levels observed in the western blot (Figure 2A). These findings confirm that GCase variants not only lead to reduced protein levels and/or activity but also alter interaction with LIMP‐2 and therefore affect subcellular localization of GCase in patient fibroblasts.

### 2.3 A Soluble LIMP‐2 Construct Facilitates Secretion and Purification of a LIMP‐2/GCase Complex from Medium of HEK 293F Cells

**Figure 3:**
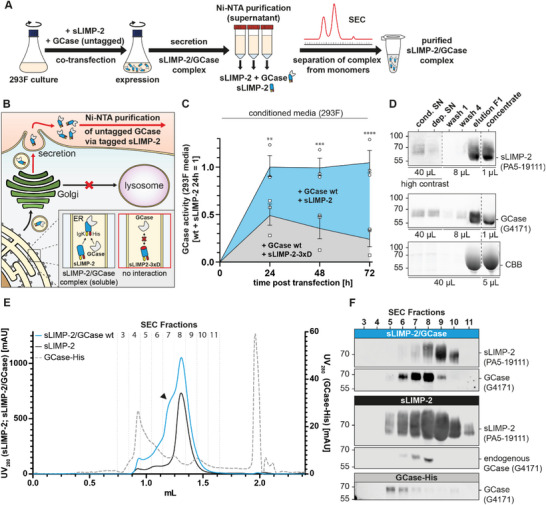
Purification of GCase utilizing soluble (s) LIMP‐2. A) Workflow for the purification of sLIMP‐2/GCase complex from HEK 293F cells. A culture is transfected to co‐express sLIMP‐2 and GCase. After 96 h, the conditioned medium is collected and Ni‐NTA purification of His‐tagged proteins is performed, yielding sLIMP‐2 and sLIMP‐2/GCase complex. Elution fractions are concentrated and separated via SEC, yielding purified sLIMP‐2/GCase protein complex. B) Cartoon of intracellular mechanisms of interaction and secretion of GCase with the soluble sLIMP‐2 construct. A soluble sLIMP‐2/GCase complex is formed in the ER. The IgK leader sequence on sLIMP‐2 then facilitates secretion of the LIMP‐2/GCase complex instead of sorting GCase to the lysosome. In comparison, the sLIMP‐2‐3xD control construct does not bind GCase and therefore does not facilitate secretion of GCase. C) GCase activity in the supernatant of HEK 293F cells overexpressing wt GCase along either sLIMP‐2 or sLIMP‐2‐3xD (n  =  3; individual transfections). Samples were taken 0‐72 h after transfection. In co‐expression with sLIMP‐2, GCase activity is higher and stable over the course of the measurements, whereas activity in the control is lower and diminishes over time. The statistical significance indicated (*) represents a comparison between both sample groups at a given time point. D) Representative western blot of analytical samples from Ni‐NTA purification of the sLIMP‐2/GCase complex from HEK 293F supernatant. Parts of the blot are shown with increased contrast to visualize faint signals. Splicing is indicated by a dashed line. Abbreviations: cond. SN: conditioned supernatant; dep. SN: depleted supernatant; wash 1/4: flow‐through of washing steps 1/4 (4 total); elution F1: elution fraction 1 (5 total). Elution of protein from Ni‐NTA resin yielded sLIMP‐2 and GCase. E) Representative SEC profiles of samples from Ni‐NTA purification of sLIMP‐2/GCase (blue), sLIMP‐2 (black), and His‐tagged GCase (grey, dashed) using a Superdex 200 Increase 3.2/300 column. sLIMP‐2 and sLIMP‐2/GCase samples share a peak at fraction 8 corresponding to the sLIMP‐2 monomer. An additional peak in fraction 7 is visible in the sLIMP‐2/GCase sample, corresponding to the sLIMP‐2/GCase complex. His‐tagged GCase runs as a major peak in fractions 4/5 with training smaller peaks in later fractions. F) Western Blot analyses of SEC fractions (corresponding to Figure 3E). Fractions of the sLIMP‐2/GCase sample show strong GCase and LIMP‐2 signals with maxima at fractions 7/8 and 8/9 respectively. Fractions of sLIMP‐2 show the same distribution with a weak GCase signal. His‐tagged GCase was most abundant in fractions 5/6. Statistics: replicates (dots, squares) with mean (line) ± SEM (C). Tests: Two‐way ANOVA with Sidak's multiple comparison test (C). ** p < 0.01, *** p < 0.001, **** p < 0.0001.

Purification of recombinant GCase disease variants for in vitro analyses exhibits challenges, starting with the introduction of an affinity tag for the pulldown of the protein. Since the N‐terminal part of GCase undergoes post‐translational cleavage,^[^ ^49^ ^]^ a C‐terminal tag is preferable. However, other studies have successfully modified both termini with affinity tags.^[^ ^50^, ^51^ ^]^ In our experimental setup however, the introduction of a C‐terminal His‐ or Strep‐tag negatively impacted the expression and activity of recombinant GCase wt in HEK 293T cells in direct comparison to the untagged enzyme (Figure S4A–C, Supporting Information). Thus, an affinity‐tag in combination with mutations additionally impacting protein stability such as the GD‐/PD‐associated variants E326K, N370S, and L444P^[^ ^21^ ^]^ analyzed here could lead to an additive negative impact on the function and stability of the enzymes as well as protein yield. Conventional overexpression of GCase makes use of an oversaturation of the cellular LIMP‐2‐dependent lysosomal transport causing passive leakage of GCase into the cell culture medium.^[^ ^35^ ^]^ For efficient large‐scale protein purification independent of an affinity tag on GCase and to exploit the stabilizing effects of LIMP‐2, we developed a co‐purification protocol of both proteins as a soluble complex (**Figure** **3**A). For this, we conceptualized a novel approach utilizing a recombinant LIMP‐2 shuttle. This construct comprises the luminal domain (aa 36–431) of LIMP‐2 linked to an *N*‐terminal immunoglobulin kappa (Ig*κ*) secretion sequence as well as a C‐terminal, TEV‐cleavable His‐tag for Ni‐NTA purification (Figure S4D, Supporting Information). This soluble LIMP‐2 (sLIMP‐2) construct still binds GCase in the ER but due to the Ig*κ* secretion sequence shuttles the LIMP‐2‐GCase protein complex toward the secretory pathway, consequently leading to secretion into the cell culture supernatant (Figure 3B). A non‐GCase‐binding sLIMP‐2 variant (sLIMP‐2‐3xD), analog to the FL‐LIMP‐2‐3xD construct, served as control. Transient expression of sLIMP‐2 together with GCase in HEK 293F suspension cells resulted in the secretion of the LIMP‐2/GCase protein complex into the culture medium. This was observed by an increase in GCase activity in the cell culture medium after co‐expression with sLIMP‐2 when compared to sLIMP2‐3xD (Figure 3C), thereby showing that active secretion of GCase via sLIMP‐2 was more efficient than passive leakage. Ni‐NTA pulldown from the cultured media yielded recombinant sLIMP‐2 with large amounts of bound GCase (Figure 3D). Untagged GCase alone showed no affinity towards the Ni‐NTA matrix (Figure S4E, Supporting Information, right side), confirming purification of a sLIMP‐2/GCase complex. Size exclusion chromatography (SEC) was used for further purification and separation of the complex from sLIMP‐2 monomers (Figure 3E). The complex of LIMP‐2 and GCase (blue line) elutes in different fractions compared to the individually expressed and purified sLIMP‐2 (black line) and His‐tagged GCase (grey line) (Figure 3E). The shift in the SEC elution profile (Figure 3E,F) indicates the interaction of both proteins and purification of a stable complex, which could be confirmed by western blot analyses (Figure 3F) and activity assays (Figure S4F, Supporting Information) of the SEC fractions, which reflect the same shift. The high shuttling efficiency of our sLIMP‐2 protein construct is also demonstrated by the presence of endogenous GCase in samples that overexpressed sLIMP‐2 only (Figure 3F, fourth blot from the top).

These findings confirm that the sLIMP‐2‐shuttle efficiently binds GCase intracellularly and transports untagged GCase into the culture medium, from which the sLIMP‐2/GCase complex can be purified. After successful purification of the sLIMP‐2/GCase wt complex, we investigated whether our newly established sLIMP‐2/GCase co‐expression system can be utilized to purify the disease‐associated GCase variants E326K, N370S, and L444P. For this, co‐expression and co‐purification of untagged GCase variants with sLIMP‐2 were performed as for GCase wt. Western blot analysis of the Ni‐NTA pulldown samples showed co‐purification of all variants along sLIMP‐2 (Figure S4G, Supporting Information). The yield of N370S GCase protein was comparable to the wt while the yield of E326K was slightly reduced. For the L444P variant however, only low amounts of GCase were co‐purified, indicating reduced protein stability or transport, which has been described before^[^ ^23^, ^48^ ^]^ and underlines our cellular data (Figures 1 and 2). Thus, this variant was not further analyzed via SEC. Co‐purified samples of E326K and N370S GCase produced SEC profiles (Figure S4H, Supporting Information) and protein distribution (Figure S4I, Supporting Information) similar to wt GCase, indicating proper interaction with LIMP‐2, formation of a stable protein complex and secretion into the cell culture supernatant.

### 2.4 A LIMP‐2‐Derived Peptide Activates GCase wt and E326K

**Figure 4:**
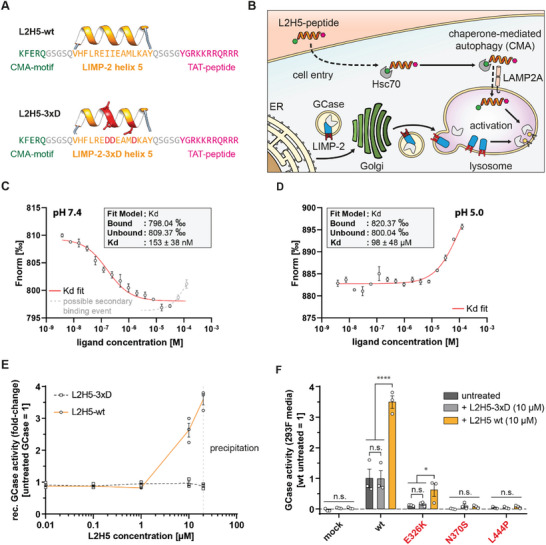
Design and effect of LIMP‐2‐derived helix 5 peptide on GCase function. A) Sequence of custom LIMP‐2 peptides. The peptides comprise the wt or 3xD variant of helix 5 of LIMP‐2 (orange), flanked by an N‐terminal lysosomal KFERQ sequence for lysosomal targeting (green) and a C‐terminal TAT‐peptide for cell penetration (pink). Linker regions are indicated in grey. B) Cartoon of uptake of LIMP‐2‐derived peptide into cells and the lysosome, where the peptide interacts with GCase, boosting its lysosomal function. C,D) Interaction of L2H5‐wt and GCase‐His at cytosolic (C) and lysosomal (D) pH as determined by MST (n  =  3 sample preparations per condition). Dots represent mean ± SEM. With increasing concentration of ligand (L2H5‐wt), changes in the MST signal (FNorm) could be observed at both conditions, indicating binding to GCase. Fitting of a Kd model (red line) yielded estimated affinities in the nanomolar range for pH 7.4 and micromolar range for pH 5.0. At pH 7.4, higher L2H5 concentrations lead to a second change in the MST signal, hinting toward a second binding event with lower affinity (illustrated as a grey dashed line). Grey datapoints were disregarded for the Kd model fit (red line). E) Enzyme activity of Cerezyme in the presence of varying concentrations of L2H5‐wt or −3xD peptides (n  =  3; individual experiments). An activating effect of L2H5‐wt was first observed in the micromolar range and increased further with peptide concentration. The addition of 10 µM of L2H5 led to a 2.63 ± 0.22‐fold increase of GCase activity. At peptide concentrations above 20 µM, precipitation of the peptide occurred as indicated by a dashed grey line. F) Effect of L2H5 peptides on the activity of recombinant GCase in conditioned HEK 293F media after overexpression of GCase variants (n  =  3, individual experiments). The activity of wt GCase and E326K were increased in the presence of 10 µM L2H5‐wt. The activity of N370S and L444P were unaffected. Statistics: mean (dot) ± SEM (C,D), replicates (dots, squares) with mean (line) ± SEM (E); Mean (column) ± SEM (F). Tests: non‐linear regression Kd model (C,D); two‐way ANOVA with Tukey's multiple comparison test (F). * p < 0.05, **** p < 0.0001, n.s.: not significant.

After observing an activating effect of LIMP‐2 on GCase wt and E326K using both recombinant proteins and HEK 293 cell models (Figure S1C,F, Supporting Information), we optimized a custom peptide to mimic LIMP‐2 binding in order to test its activating potential on the different GCase variants. Considering previous biochemical data about the LIMP‐2 and GCase interaction^[^ ^42^ ^]^ and structural data from the single proteins,^[^ ^39^, ^43^ ^]^ we designed a peptide comprising helix 5 of LIMP‐2 for GCase interaction, flanked by an N‐terminal KFERQ motif to provoke chaperone‐mediated autophagy (CMA), and a C‐terminal transactivator of transcription (TAT) sequence for cell penetrance. We termed this peptide L2H5‐wt (**Figure** **4**A). An anologous peptide harboring the 3xD mutations (Figures S1D,S4A, Supporting Information) was designed as a non‐binding control^[^ ^42^ ^]^ and termed L2H5‐3xD. We expected the TAT and KFERQ motifs to work in tandem for the peptide to enter the cell and then be recognized and targeted specifically to the lysosome by Hsc70, thereby not interfering with LIMP‐2 mediated transport of GCase from the ER to the lysosome (see Figure 4B).

Interaction of the L2H5‐wt peptide with recombinant GCase was assessed via MST at cytosolic and lysosomal pH (Figure 4C,D). For this, we used His‐tagged GCase wt that we purified in‐house from HEK 293F cells. At cytosolic pH (pH 7.4), we observed interaction between L2H5‐wt and GCase starting at nanomolar concentrations (Figure 4C). Interestingly, the two highest L2H5‐wt concentrations (grey data points) again led to a small change in the MST signal, hinting toward an additional low‐affinity binding event (Figure 4C, indicated by a grey dotted line). However, due to limitations in the solubility of the peptide, this secondary binding event could not be investigated further. We therefore disregarded the last two data points for the Kd model fit and obtained a Kd value of 153 ± 37 nm for the primary, high‐affinity binding event (Figure 4C, red line). At lysosomal pH (pH 5.0), the interaction could still be observed but affinity was substantially lower (Figure 4D). We estimated a Kd value of 98 ± 48 µm but could not experimentally reach full binding saturation due to insufficient solubility of the peptide at higher concentrations (Figure 4D, red line). We then tested the effect of L2H5‐wt on GCase activity using a recombinant enzyme (Cerezyme/imiglucerase) and 4MU as a substrate. With the addition of L2H5‐wt to the reaction mixture, we observed a concentration‐dependent increase of substrate turnover starting in the low micromolar range (Figure 4E), which is similar to our observations using MST (Figure 4C,D). As expected, the control peptide L2H5‐3xD showed no effect on GCase activity (Figure 4E). Precipitation of the L2H5‐wt peptide was observed at high concentrations (>20 µm) in the 4MU assay buffer.

As a follow‐up, we investigated whether disease‐associated variants of GCase are susceptible to activation by L2H5‐wt. For this, we expressed untagged GCase (wt, E326K, N370S, L444P) in HEK 293F cells. Due to the oversaturation of endogenous LIMP‐2, GCase variants escaped into the culture media, which we utilized for activity analyses. For this, we spiked 10 µm of L2H5‐wt or L2H5‐3xD into the cell culture supernatants and measured two to threefold increased enzymatic activities for GCase wt and E326K (Figure 4F). For GCase variants N370S and L444P, no increase in activity could be measured (Figure 4F). After samples were concentrated, western blot analysis of the conditioned media confirmed the presence of all GCase variants, except for L444P GCase, again suggesting low stability and/or ER retention of this variant (Figure S5A, Supporting Information).

These findings confirm that helix 5 of LIMP‐2 binds to a potent allosteric activation site of GCase, which at the same time is important for the transport of GCase to the lysosome by LIMP‐2. However, by targeting the helix 5 peptide directly to the lysosome, interference with GCase transport could be avoided, thus restricting interaction and activation to the lysosome (Cartoon Figure 4B). To test this hypothesis and the effect of the L2H5‐peptides in a disease environment, they were next applied in PD patient‐derived fibroblasts.

### 2.5 L2H5‐wt is Delivered to the Lysosome and Rescues Lysosomal GCase Activity in PD Patient‐Derived Fibroblasts Harboring the E326K Variant

**Figure 5:**
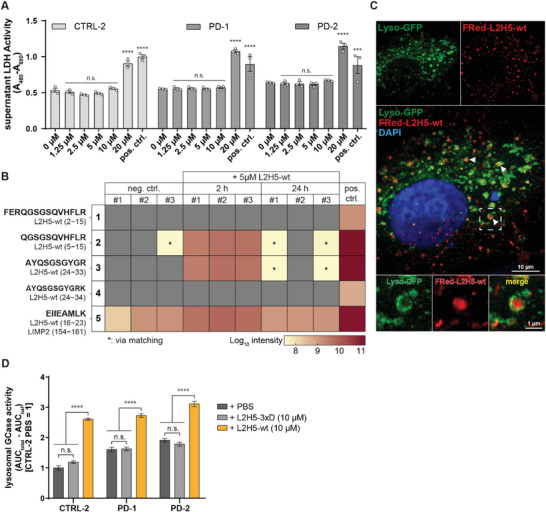
Uptake and effect of L2H5 peptides on control and PD fibroblasts. A) Assessment of cell death via LDH activity in culture medium in CTRL‐2, PD‐1, and PD‐2 fibroblast lines (n  =  3; wells from the 96‐well plate). Cells were treated with varying concentrations of L2H5‐wt peptide for 72 h. concentrations up to 10 µM did not lead to an increase in cell death. At a concentration of 20 µM however, LDH activity in the medium was significantly increased, indicating increased cell death due to treatment. Effects were comparable between all three lines. Significance is shown in comparison to the 0 µM data group for each cell line respectively. B) Presence of tryptic exogenous L2H5‐wt and endogenous LIMP‐2‐derived peptides in LE fractions of HEK293T cells after treatment with PBS (neg. ctrl.) or 5 µM L2H5‐wt for 2 h and 24 h. Determined via mass spectrometry. The positive control represents a sample spiked with 0.5 µg of L2H5 before analysis. L2H5‐wt‐specific peptides 1‐4 were detected in the pos. ctrl (all 4) and the cells treated with L2H5‐wt for 2 h (peptides 2 and 3). Low amounts of peptides 2 and 3 were also detected at the 24 h time point, but only by matching (indicated with an asterisk) and only in two of the three samples. Peptide 5, which is a tryptic product of both L2H5‐wt and endogenous LIMP‐2, was detected in all samples as expected, with higher abundance in the spiked control and the 2 h treated samples. C) Representative immunofluorescence image of CTRL‐2 fibroblasts with GFP‐labeled lysosomes (CellLight Lysosomes‐GFP) after 2 h of treatment with 0.25 µg µL^−1^ FRed‐L2H5‐wt. Objective magnification: 63x. Top: single channels. Middle: merged picture. Bottom: zoomed in single channels and merged picture of area inside a white frame. Green: GFP; red: FusionRed; blue: DAPI. FusionRed signal dots were visible within the cell. Some dots were surrounded by GFP signal located in the lysosomal membrane, thus confirming the presence of FRed‐L2H5‐wt inside the lysosome as shown by white arrows and in the zoomed‐in section. D) Live cell GCase activity in primary human control fibroblasts and PD‐patient‐derived fibroblasts harboring E326K mutations (n  =  3; wells of a 96‐well plate). The cells were treated with PBS or L2H5 peptides (wt and 3xD). The graph shows lysosomal GCase activity as an area between curves (see materials and methods). In all three cell lines, lysosomal GCase activity was dramatically boosted after treatment with L2H5‐wt. In contrast, treatment with the non‐binding L2H5‐3xD peptide did not affect lysosomal GCase activity. See Figure S5E (Supporting Information) for individual activity graphs with replicates. Statistics: replicates (dots) with mean (column) ± SEM (A); mean (column) ± SEM (D). Tests: Two‐way ANOVA with Dunnett's multiple comparison test (A); Two‐way ANOVA with Tukey's multiple comparison test (D). * p < 0.05, *** p < 0.001 **** p < 0.0001, n.s.: not significant.

As the L2H5‐wt peptide showed an activating effect not only on GCase wt but also on the E326K variant, we explored the effects of peptide treatment on primary human fibroblasts (CTRL‐2, PD‐1, and PD‐2). First, we investigated whether L2H5‐wt is tolerated by cells and successfully delivered to the lysosome. For this, we treated primary human fibroblasts (CTRL‐2, PD‐1, PD‐2) with different concentrations of peptide (1.25–20 µm) for 72 h and measured cell toxicity via LDH release (**Figure** **5**A). Concentrations up to 10 µm were tolerated well by the cells, whereas 20 µm lead to a strong increase in LDH release. We therefore chose concentrations between 5–10 µm for subsequent treatment experiments.

Next, we treated HEK 293T cells with 5 µm of peptide for 2 and 24 h, generated LE fractions and performed mass spectroscopy to identify L2H5‐wt‐specific peptides in the lysosome (Figure 5B). We detected five unique tryptic peptides in the spiked positive control. Peptides 1–4 are specific for L2H5‐wt while peptide 5 is also a tryptic peptide of LIMP‐2. Expectedly, peptide 5, which is a digestion product of both L2H5‐wt and endogenous LIMP‐2, was detected in all samples with the highest abundance in the 2 h sample and the spiked control. L2H5‐wt‐specific peptides 2–4, which were the most abundant in the spiked control, could be detected by mass spectrometry 2 h and by matching 24 h after treatment, with abundance after 24 h being substantially lower compared to the 2 h time point. Peptides 1 and 4 were the rarest tryptic peptides, with only small amounts detected in the spiked control. These peptides could not be detected in the samples after treatment, likely due to their low abundance.

To visualize the uptake of L2H5‐wt, we designed an L2H5‐wt peptide fused to FusionRed (Figure S5B, Supporting Information), which we expressed in E. coli and purified using Ni‐NTA purification and SEC (see Figure S5C,D, Supporting Information for quality control). We then transduced primary human PD‐1 fibroblasts with CellLight Lysosomes‐GFP to label lysosomes and treated the cells with 0.25 µg µL^−1^ of FRed‐L2H5‐wt for 2 h before fixation. Using confocal microscopy, we could visualize subcellular FRed signal in a dot‐like distribution throughout the cells, indicating uptake into endocytic vesicles (Figure 5C). Some dots were visibly located within GFP‐labeled lysosomes (Figure 5C white arrows and zoomed in the section indicated by white frame), confirming successful lysosomal delivery of FRed‐L2H5‐wt.

Finally, we measured live cell lysosomal GCase activity in CTRL‐2, PD‐1, and PD‐2 lines, after treatment with PBS (control), 10 µm of L2H5‐wt or 10 µm of L2H5‐3xD for 48 h. Compared to PBS‐treated cells, lysosomal GCase activity increased significantly in all three lines after treatment with L2H5‐wt, indicating activation of GCase within the lysosome (Figure 5D; Figure S5E, Supporting Information). In turn, treatment with the non‐binding L2H5‐3xD peptide did not elevate lysosomal GCase activity (Figure 5D; Figure S5E, Supporting Information).

Altogether, this data shows that a peptide mimicking helix 5 of LIMP‐2 is taken up by primary fibroblasts and successfully delivered to the lysosome. Inside, L2H5‐wt increases the lysosomal enzymatic activity of GCase in both control and patient‐derived cells. This serves as a proof‐of concept that targeting the LIMP‐2 interaction site on the GCase protein (by a peptide or potentially also a small compound) could serve as a treatment strategy to rescue GCase activity, especially for the PD‐associated E326K variant.

## 3 Discussion

In this study, we investigated the interaction of the GD‐ and PD‐associated lysosomal hydrolase GCase and its transporter, the lysosomal membrane protein LIMP‐2. Using a combination of HEK cell lines, patient‐derived fibroblasts and recombinant protein engineering, we provide evidence that LIMP‐2, in addition to being a transporter of GCase, functions as a chaperone and allosteric activator of GCase. Upregulation of LIMP‐2 via transient co‐expression with different GCase variants led to an increase in lysosomal transport and activity of GCase wt and the PD‐associated GCase E326K in HEK 293T cells. In vitro, we observed increased enzyme activity of recombinant GCase when adding a recombinant LIMP‐2 ectodomain, confirming a transport‐independent activation effect, which is in line with previous studies.^[^ ^42^ ^]^

With LIMP‐2 being the only known transporter of GCase to the lysosome, the interaction of both proteins is necessary for lysosomal function and health.^[^ ^37^, ^52^ ^]^ In addition to reduced activity or stability of disease‐associated GCase variants, insufficient lysosomal transport by LIMP‐2 can also contribute to GCase‐related pathology. This is supported by the fact that the LIMP‐2‐coding *SCARB2* gene is itself a risk factor for PD, connected via GCase and the GluCer/a‐syn axis.^[^ ^41^, ^53^, ^54^ ^]^ We therefore investigated whether the three common GCase variants E326K, N370S and L444P exhibited altered binding to LIMP‐2. Overexpression of FL‐LIMP‐2 along GCase variants in HEK293T cells led to increased protein levels of wt, E326K, and N370S in LE fractions (Figure 1D,E), suggesting interaction with LIMP‐2 and transport of these variants. For the L444P variant, however, this effect was not observed. FL‐LIMP‐2 expression levels in both whole‐cell lysate, as well as LE fractions, were reduced in co‐expression with L444P GCase, possibly indicating a negative effect on the ER by this variant. Previous studies have shown that L444P is retained in the ER and activates the unfolded protein response.^[^ ^48^ ^]^ Thus, reduced levels of FL‐LIMP‐2 could be explained by variant‐induced ER stress and/or co‐degradation FL‐LIMP2 while bound to the L444P GCase variant. FL‐LIMP‐2 levels in co‐overexpression with E326K GCase appeared reduced as well but did not reach significance, and negative effects on the ER have are unknown for the E326K variant to date. The N370S variant was found enriched in the lysosomal fraction after LIMP‐2 overexpression, but its enzymatic activity did not increase. This indicates that the functional deficit of the N370S variant is not due to impaired lysosomal transport, but to a compromised enzymatic activity that cannot be rescued by LIMP‐2. Considering that the N370S variant is classified as a mild variant,^[^ ^55^ ^]^ we were surprised to find that the activity of N370S in our experiments was highly impaired to an extend comparable to the L444P variant. However, other studies measured the activity of certain variants and found that the activity of N370S toward 4MU was reduced similarly to L444P.^[^ ^22^, ^23^ ^]^ N370S is a functionally impaired, yet stable variant compared to the non‐active, but unstable L444P variant, possibly explaining the difference in pathogenicity.

To investigate the LIMP‐2/GCase interaction in a disease‐relevant context further, we characterized primary fibroblast cell lines from three controls, two PD patients harboring a *GBA^wt/E326K^* genotype and a GD patient with a *GBA^L444P/L444P^* genotype. The PD fibroblasts showed reduced GCase activity compared to controls, confirming our findings from the overexpression studies that the E326K variant is impaired in its activity. In comparison, the GD fibroblasts expectedly showed very low GCase levels and almost completely abolished activity. To investigate the interaction of GCase with LIMP‐2 in these cells, we performed immunofluorescence and co‐localization analysis of GCase and LIMP‐2 via Pearson's correlation coefficient. Compared to control lines, both PD lines showed mildly reduced co‐localization of LIMP‐2 and GCase while co‐localization was strongly reduced in the GD line. The latter also showed highly reduced GCase signal intensity, which corresponds to the low expression levels observed via western blot. Along with the results from overexpression studies, these findings show that E326K GCase and L444P GCase are impaired in their interaction with LIMP‐2, with the impairment being mild for E326K and strong for L444P. This matches with a recently published study that performed interactome analysis for wt, E326K, and L444P GCase and found that interaction between LIMP‐2 and E326K GCase was moderately reduced while interaction with the L444P variant was almost abolished.^[^ ^56^ ^]^

In addition to our cellular data, we designed a soluble, tagged LIMP‐2‐shuttle that facilitates secretion of a sLIMP‐2/GCase transport‐complex into the cell supernatant of HEK 293F cells. By performing Ni‐NTA pulldown of His‐tagged sLIMP‐2, we showed that while all GCase variants were able to bind to the shuttle and be secreted from the cell, co‐pulldown efficiency varied drastically between variants. N370S was pulled down with similar efficiency to the wt. E326K showed slightly reduced pulldown efficiency and co‐pulldown of the L444P variant yielded only small amounts of GCase (Figure S4F, Supporting Information). These findings are again consistent with our previous experiments in HEK293T cells (Figure 1). Furthermore, we could confirm the integrity of the sLIMP‐2/GCase complex after purification for wt, E326K, and N370S GCase via SEC. We conclude from this that our sLIMP‐2‐based shuttle system is a suitable approach for the purification and analysis of the LIMP‐2/GCase complex, allowing large‐scale production of the recombinant complex for either functional or structural analysis. In addition, this system circumvents the need of modifying GCase with an affinity tag for purification. In our hands, a C‐terminal His‐tag negatively affected expression in comparison to untagged GCase. Although other studies have successfully purified modified GCase enzymes either via a C‐terminal His‐tag^[^ ^50^ ^]^ or via an N‐terminal His‐tag with a modified signal peptide,^[^ ^51^ ^]^ introducing such protein modifications may affect maturation/biological function and could thus distort the impact of single point mutations on GCase.

Overall, we consider the here presented purification strategy ideal to study structural characteristics of the LIMP‐2/GCase complex as well as native GCase structures, especially from GCase variants impaired in their stability or maturation.^[^ ^21^ ^]^ This protein purification protocol enables the physiological formation of the complex within the ER, preserving post‐translational modification steps along the secretory pathway, which seem to be important for the stability of the complex. Further, this permits pharmacological screenings in order to identify GCase modifiers. Importantly, GCase‐binding small molecules identified by compound screenings on GCase in complex with LIMP‐2 will not interfere with lysosomal transport of the enzyme.

With LIMP‐2 being able to bind and activate GCase wt and the E326K variant, we investigated whether we can exploit this interaction further. For this, we designed a custom helical peptide derived from helix 5 of the LIMP‐2 ectodomain. The helix 5 motif was identified as a potential interaction motif in binding GCase before by biochemical analyses and the resolution of the LIMP‐2 crystal structure.^[^ ^39^, ^42^ ^]^ Based on this data, the LIMP‐2‐derived helix 5 peptide was flanked with a CMA‐ and TAT‐motif in order to facilitate cell entry and lysosomal uptake. In in vitro studies, this peptide was capable of binding GCase and boosting the activity of recombinant GCase wt and E326K, confirming that helix 5 of LIMP‐2 alone is able to mediate interaction with GCase. Further, the LIMP‐2 peptide was applied to control fibroblasts as well as fibroblasts of two PD patients, both harboring a heterozygous *GBA‐E326K* risk variant. We confirmed uptake of the peptide into the lysosomes by mass spectrometry and immunofluorescence (Figure 5B,C). Strikingly, treatment of cells with this newly engineered peptide at a concentration of 10 µm resulted in a significant increase in lysosomal GCase activity (Figure 5D) without causing cell toxicity (Figure 5A). Our study therefore reveals a novel mechanism of GCase regulation via LIMP‐2 and uncovers the LIMP‐2 interaction site on GCase as a potential target for therapeutic intervention in GD and PD. However, affinity of the L2H5‐wt peptide toward GCase was reduced under acidic conditions as shown by MST measurements (Figure 4C,D), highlighting the importance of investigating target engagement at cytosolic and lysosomal conditions as well as optimization of therapeutic compounds to ensure interaction in the correct subcellular compartments.

In the context of diseases, where lysosomal GCase activity is reduced, activation of either impaired mutated GCase or residual wt GCase could provide a therapeutic strategy for restoring lysosomal function. Both in GBA1‐associated and in GBA1‐wt PD models and patients, ongoing preclinical and clinical studies are already exploring this possibility, predominantly at the stage of screening for GCase activators for the treatment of Gaucher and Parkinson's disease.^[^ ^57^, ^58^ ^]^ Despite the expected increase of the prevalence of PD and worldwide disease burden due to increasing life expectancy and environmental influences, none of the currently approved PD therapies slows disease progression.^[^ ^59^ ^]^ After a multitude of previous clinical trials including alpha‐synuclein lowering strategies failed to modify the disease course, GCase has recently become one of the major targets in drug development.^[^ ^58^, ^60^ ^]^ The present study identifies the potential of the LIMP‐2 binding site on GCase to be exploited as an allosteric activation site that is susceptible to small ligands. However, it must not be disregarded that this site is crucial for the lysosomal transport of GCase. Any therapeutic approach targeting the LIMP‐2 interaction site would need to be specifically delivered to the lysosome to not disrupt the lysosomal transport of GCase via LIMP‐2. While we did not observe this in our experiments, the possibility of disrupting transport should be considered and thoroughly investigated for all potential GCase activators. When treating cells with the LIMP‐2‐derived peptide, we observed cell toxicity on primary fibroblasts starting at a concentration of 20 µm, indicating a possible overdosing effect that could potentially be related to side effects. Therefore, further studies are necessary in order to investigate possible off‐target interactions of this LIMP‐2‐mimicking approach, as LIMP‐2 appears to have additional functions besides the transport of GCase, such as a suggested role in cholesterol transport or as a cell surface receptor.^[^ ^61^, ^62^, ^63^ ^]^ Furthermore, not all GCase variants are susceptible to activation via LIMP‐2. In our study, only the wt and the PD‐associated E326K variants, but not the GD and PD‐associated N370S and L444P variants were responsive to modulation via LIMP‐2. This means that for GD cases, where both GBA1 alleles are affected and cells have no GCase that can be rescued by LIMP‐2, a LIMP‐2‐derived approach is likely, not feasible. Additionally, recent research has shifted from the strictly loss‐of‐function oriented role of GCase in PD and shed light on possibly pathological gain‐of‐function effects induced by specific GCase variants. These include ER sequestration and stress,^[^ ^31^ ^]^ lipid droplet accumulation,^[^ ^33^ ^]^ disruption of CMA,^[^ ^32^ ^]^ and disturbance of mitochondrial function.^[^ ^56^ ^]^ Therefore, simply increasing GCase activity in the lysosome may not be the only possible or necessary approach to therapeutically target GCase in PD. The previously failed clinical trial for venglustat (ClinicalTrials.gov ID NCT02906020), a compound that inhibits the synthesis of glucosylceramide, showed that changing GluCer levels alone may not be sufficient to positively affect disease progression.^[^ ^64^, ^65^ ^]^

In conclusion, we demonstrate that LIMP‐2 is not only a transporter, but also a chaperone and key modulator important for proper GCase function. Our study provides a strategy for how this feature could be exploited to increase the lysosomal activity of GCase and rescue GCase deficiency in a disease context. Recent data have postulated that interaction between LIMP‐2 and GCase is abolished once the transporting complex reaches the acidic environment of the lysosome.^[^ ^66^, ^67^ ^]^ However, we show that LIMP‐2 increases GCase activity under acidic conditions and that a LIMP‐2‐mimicking peptide can bind and increase GCase activity within lysosomes of PD patient‐derived fibroblasts. Therefore, we propose that the interaction between LIMP‐2 and GCase transcends the lysosomal transporting pathway, revealing a potent regulatory mechanism that is targetable using small ligands. These findings as well as our LIMP‐2/GCase purification protocol provide a basis and innovative tool for the design of novel GCase activators.

## 4 Experimental Section

### Expression Constructs

Strep‐tagged human GCase was expressed using a self‐generated pcDNA 3.1‐derived plasmid. All other expression constructs used for mammalian expression were based on the pCMV3 vector (Sino Biological Inc., Peking, China, #CV011). For expression of full‐length human LIMP‐2 (Sino Biological Inc., Peking, China, #HG11063‐CH), and untagged human GCase wt (Sino Biological Inc., Peking, China, #HG12038‐UT), commercially available expression constructs were used. For the expression of 10xHis‐tagged GCase wt, a commercially available plasmid (Sino Biological Inc., Peking, China, #HG12038‐CH) was modified to make the C‐terminal His tag cleavable by tobacco etch virus (TEV) protease. GCase variant expression constructs (E326K, N370S, L444P) were generated from the tagged and untagged wt plasmids (see above) via site‐directed mutagenesis. The expression construct for the secreted LIMP‐2 ectodomain (sLIMP‐2) was cloned in‐house. It comprises amino acids 36–431 of LIMP‐2 with an N‐terminal IgK leader sequence and a C‐terminal, TEV‐cleavable 10xHis tag (Figure S4D, Supporting Information). LIMP‐2‐3xD constructs were created from their respective templates (full‐length LIMP‐2 and sLIMP‐2 plasmids) via site‐directed mutagenesis introducing I155D, I156D, and L160D mutations (see Figure S2E, Supporting Information for base changes).

As a mock control for transfection, an empty pcDNA 3.1 (ThermoFisher Scientific Inc., Waltham, MA, United States, #V79020) vector containing no cDNA was used.

For the expression of FusionRed‐tagged L2H5‐wt fusion protein, an expression plasmid based on the pT7 vector was cloned in‐house (pT7‐FRed‐L2H5‐wt). The cDNA comprises an N‐terminally tagged FusionRed linked to L2H5‐wt (aa: QVHFLREIIEAMLKAYQ) via a GGGGS linker, followed by a C‐terminal TAT‐peptide (aa: GRKKRRQRRRPQ). Following plasmids were used in the cloning procedure: cDNA donor: FusionRed‐pBAD (Addgene, Watertown, MA, United States, #54 677),^[^ ^68^ ^]^ full‐length LIMP‐2 (Sino Biological Inc., Peking, China, #HG11063‐CH); backbone: pT7‐7 asyn WT (Addgene, Watertown, MA, United States, #36 046).^[^ ^69^ ^]^

### Recombinant Proteins and Peptides

For some experiments, industrially produced recombinant proteins were used. These were sLIMP‐2‐FC (Sino Biological Inc., Peking, China, #11063‐H03H), imiglucerase/Cerezyme (Sanofi Genzyme, Cambridge, MA, United States). Other recombinant proteins (sLIMP‐2, His‐tagged GCase, and untagged GCase variants) were produced in‐house using HEK 293F cells as described below (see paragraph “Generation of Recombinant Proteins”).

Custom L2H5‐wt and 3xD peptides were synthesized by BioCat (Heidelberg, Germany). Both peptides were synthesized with >95% purity, TFA removal, N‐terminal biotin modification and C‐terminal amidation. Amino acid sequences: L2H5‐wt: KFERQGSGSQVHFLREIIEAMLKAYQSGSGYGRKKRRQRRR; L2H5‐3xD: KFERQGSGSQVHFLREDDEAMDKAYQSGSGYGRKKRRQRRR.

FusionRed‐tagged L2H5‐wt protein was produced in‐house using E. coli (BL21 DE3 pLysS) as described below in the paragraph “Expression and Purification of FusionRed‐tagged L2H5‐wt Protein”.

### Cell Lines, Cultivation and Transient Overexpression

HEK 293F cells (ThermoFisher Scientific Inc., Waltham, MA, United States, #R79007) were cultivated in FreeStyle 293 expression medium (ThermoFisher Scientific Inc., Waltham, MA, United States, #12 338 018) in vented flat‐bottom Erlenmeyer flasks (ThermoFisher Scientific Inc., Waltham, MA, United States, #4115‐0125, #4115‐0250, #4115‐0500, #4115‐1000, #4115‐2000) on a MaxQ CO_2_ Plus orbital shaker (ThermoFisher Scientific Inc., Waltham, MA, United States, #88 881 102) at 125 rpm, 37 °C and 8% CO_2_. Regularly, the cell count was determined using a Cellometer Auto T4 Plus cell counter (Nexcelom Biocience LLC, Lawrence, Massachusetts, United States). The cells were maintained at concentrations of 1 × 10^5^ to 2 × 10^6^ cells and passaged by diluting the culture with fresh pre‐warmed FreeStyle expression medium. For seeding, cells were counted and the desired cell number was transferred to 50 mL tubes. The medium was removed by centrifugation (500 × *g*, 3 min, RT) and the cells were suspended in fresh FreeStyle expression medium at a concentration of 1 × 10^6^ cells per mL and vortexed for 10 s. The transfection mix was prepared as follows: For every 1 × 10^6^ cells, 1 µg of DNA (per plasmid) and polyethyleneimine (PEI MAX, 1 µg ml^−1^, Polysciences, Inc., Warrington, PA, United States, #24765‐1) in a ratio of 1:3 (DNA to PEI) were added to 50 µL (per µg of DNA) of OptiMEM (ThermoFisher Scientific Inc., Waltham, MA, United States, #31 985 070). After 25 min of incubation at RT, the transfection mix was added to the culture. Sixteen hours after transfection, a final concentration of 3.75 mm valproic acid (Sigma‐Aldrich, St. Louis, MO, United States, #P4543) was added to the culture.

HEK 293T cells (Leibniz Institute DSMZ‐German Collection of Microorganism and Cell Cultures GmbH, Braunschweig, Germany) were cultured on TC dishes (Sarstedt, Nümbrecht, Germany, #83.3902, #83.3903) in Dulbecco's modified eagle medium (DMEM, Gibco, Carlsbad, CA, United States) supplemented with 10% fetal calf serum (FCS, PANbiotech, Aidenbach, Germany) and 1× penicillin/streptomycin (Sigma‐Aldrich, St. Louis, MO, United States, #P0781) at 37 °C, 5% CO_2_ and 95% humidity. Passaging was done at 70–90% confluence by detaching with trypsin (PAN‐Biotech, Aidenbach, Germany, #P10‐023100) for 5 min and transferring part of the culture to a new dish containing fresh medium. The HEK 293T cells were transfected using PEI at 60–70% confluence. For a 10 cm TC dish, the transfection mix was prepared by adding 5 µg plasmid DNA, 15 µg of PEI (ratio of 1:3 DNA to PEI) to 300 µl DMEM without FCS. After incubation for 25 min at room temperature, the mix was added to the cells. If the cell supernatant was analyzed, the cells were washed twice with phosphate‐buffered saline (PBS, Gibco, Carlsbad, CA, United States) and the medium was changed to DMEM without FCS 16 h after transfection.

Primary human fibroblasts of controls and PD patients were derived from skin biopsies as described previously.^[^ ^70^ ^]^ Additionally, the following GD cell line was obtained from the NIGMS Human Genetic Cell Repository at the Coriell Institute for Medical Research: GM10915. Fibroblasts were cultured on TC dishes (see above) in DMEM with 15% v/v FCS, 1× penicillin/streptomycin, and 2 ng mL^−1^ FGF (R&D Systems, Minneapolis, MN, United States, #233‐FB). Twice a week, each line was either passaged when confluent or the culture medium was replaced with fresh pre‐warmed medium. The following fibroblast lines were used in this study: Control‐1: UKERf1E4‐1, Control‐2: UKERfO3H‐1, Control‐3: UKERf4CC‐1, PD‐1: UKERfG21‐1, PD‐2: UKERf3J3‐1, GD: GM10915.

Characteristics of individuals are listed in **Table** **1**. Experiments were carried out in accordance with the local Institutional Review Board approval (“Biobank to analyze biomarkers and generate human cellular models of neurological diseases”, No. 17‐259‐B, Ethics Committee of the Friedrich‐Alexander‐Universität Erlangen‐Nürnberg, Erlangen, Germany). Written informed consents were received from all control and PD participants prior to inclusion into research studies.

**Table 1:** Clinical characteristics of controls and patients.

Line	Control‐1	Control‐2	Control‐3	PD‐1	PD‐2	GD
Identifier	UKERf1E4‐1	UKERfO3H‐1	UKERf4CC‐1	UKERfG21‐1	UKERf3J3‐1	GM10915
Sex	male	male	male	male	male	male
Family history	–	–	–	positive (father: PD at age 68)	unremarkable	unknown
Age at PD onset	–	–	–	59 years	30 years	–
Age at biopsy	53 years	71 years	54 years	65 years	65 years	7 years
Hoehn & Yahr stage at biopsy	–	–	–	2	4	–
Presenting symptom	–	–	–	bradykinesia of the right arm	tremor of the right leg	Gaucher disease
Non‐motor symptoms	–	–	–	urinary urgency, hyposmia	REM sleep behavioral disorder, depression, hyposmia	Hepatosplenomegaly, hearing loss
Levodopa response	–	–	–	positive	positive	–
*GBA1* variants	None	None	None	rs2230288/ E326K heterozygous	rs2230288/ E326K heterozygous	rs421016/L444P homozygous

All cell lines used in this study were regularly tested and confirmed negative for mycoplasma using a MycoAlert Mycoplasma Detection Kit (Lonza, Basel, Switzerland, #LT07‐318). All lines were kept at a passage number below 20 for all experiments.

### Genomic Analyses

For each of the three control and two PD fibroblast cell lines, genomic DNA was isolated and analyzed for variants in the coding sequence and potential splice mutations at the intron‐exon boundaries of the *GBA1* gene. The analysis was performed via a whole exome sequencing approach. For massive parallel sequencing, genomic DNA of each cell line was processed according to the Illumina DNA Prep protocol (Illumina, Inc., San Diego, CA, USA). Library quantification was carried out with the High Sensitivity DNA Kit on a Bioanalyzer (Agilent Technologies, Böblingen, Germany) and the QubitdsDNA HS Assay Kit (Life Technologies, Darmstadt, Germany). The Library was sequenced as a 100 bp paired‐end run on a NextSeq2000 system (Illumina, Inc., San Diego, CA). All reads were aligned to the human reference genome (UCSC hg 19, NCBI build 37.1) and variant detection was performed with the JSI medical systems SEQNEXT module (JSI medical systems GmbH, Ettenheim, Germany).

For both PD patients, exome sequencing revealed the same heterozygous variant within the *GBA1* gene (NM_000157.4): c.1093G > A; p.Glu365Lys, rs2230288 (Figure S2, Supporting Information). This variant was also known and referred to as E326K in this paper. No other variants than those two were found in the *GBA1* gene for the five tested cell lines. For the GD line (GM10915) obtained from Coriell, the authors relied on the genetic information provided and did not perform additional genomic analysis. This line was homozygous for c.1448T > C; p.Leu483Pro, rs421016, referred to as L444P.

### Toxicity Assessment of Cell Treatment via LDH Release

Primary human fibroblasts were seeded on a 96‐well plate at 3 × 10^4^ cells per well. The next day, the medium was replaced with a 50 µL fresh fibroblast medium containing various concentrations of L2H5‐wt peptide (0–20 µm). The cells were incubated for 72 h before the conditioned medium was harvested. LDH release to the cell medium as a marker for cell death was measured using a CyQuant LDH Cytotoxicity Assay Kit (Invitrogen, Waltham, MA, United States #C20300) following the manufacturer's instructions. For each condition, three wells were seeded and measured as triplicates. A_480_ and A_680_ absorption values were measured using a ClarioStar (BMG LABTECH, Ortenberg, Germany) multiplate reader. LDH enzyme activity was determined by calculating the difference between A_480_ and A_690_ values for each well.

### Cell Lysis

Harvested cells were washed three times with cold PBS and pelleted (1.000 × g, 3 min, 4 °C). The PBS was discarded and 50–200 µL of GCase activity buffer (GCase activity buffer, 0.15 m phosphate/citrate buffer + 0.25% w/v sodium taurocholate (Sigma‐Aldrich, St. Louis, MO, United States, #T9034)) + 0.25% v/v Triton X‐100 (Carl Roth, Karlsruhe, Germany, #3051.2)) + 1× cOmplete protease inhibitor cocktail (PIC, Roche, Penzberg, Germany, #11 697 498 001) were added, followed by incubation on ice for 60 min. After the incubation, the samples were centrifuged (15 000 × *g*, 15 min, 4 °C) and the supernatant was collected. Protein concentrations were determined using Pierce BCA Protein Assay Kit (ThermoFisher Scientific Inc., Waltham, MA, United States, #23 227).

### Enrichment of Lysosomes from HEK 293T Cells

For lysosomal enrichment, HEK 293T cells were seeded on two 15 cm TC dishes and transfected with GCase and LIMP‐2 variants at 60–90% confluence as described before. Forty‐eight hours after transfection, cells were harvested and washed twice with PBS. After centrifugation (500 × *g*, 5 min, 4 °C), PBS was removed and 600 µL of sucrose HEPES buffer (250 mm sucrose, 10 mm HEPES, 100 mm EDTA, pH 7.4) were added to each cell batch. A small sample of cells was taken from each batch to prepare whole cell lysates. The remaining cells were then homogenized at 300 rpm for 60 s using a cell homogenizer (Glas‐Col, Terre Haute, USA), followed by centrifugation (6800 × *g*, 5 min, 4 °C) to pellet cell debris. The supernatant was collected and centrifuged once more (17 000 × *g*, 10 min, 4 °C). The resulting pellet was dispersed in 50 µL of GC AA buffer, followed by a 10 min incubation on ice before centrifugation (20 000 × *g*, 5 min, 4 °C) and collection of the supernatant (LE fraction). After quantification of protein concentrations via BCA (see above), 2.5 µg of protein was used for a GCase activity assay and 10 µg was used for western blot analysis. Both whole‐cell lysates as well as lysosomal fractions were analyzed.

### Purification of LIMP‐2/GCase Complex from Conditioned 293F Medium

A large culture (200–500 mL) of HEK 293F cells was prepared and co‐transfected with sLIMP‐2 (or 3xD control) and a GCase expression construct (wt, E326K, N370S or L444P) as described above. Expression was carried out for a total of 96 h. The conditioned cell supernatant was harvested by centrifugation (1500 × g, 10 min, 4 °C) to remove cells and debris, followed by filtration using a 0.22 µm pore size filter unit (Carl Roth, Karlsruhe, Germany, #P666.1). Purification of His‐tagged proteins was carried out in a batch approach. For every 100 mL, a bed volume (BV) of 75 µL of Ni‐NTA beads (MACHEREY‐NAGEL GmbH and Co. KG, Düren, Germany, #745 400) was added to the conditioned medium. The mixture was agitated overnight (4 °C) before spinning down the beads (500 × g, 5 min, 4 °C). The beads were washed 5 times with 10× BV of wash buffer (50 mm NaH_2_ PO_4_, 300 mm NaCl, 20 mm imidazole (Merck Millipore, Billerica, MA, United States, #814 223), pH 8.0) before eluting 5 times with 1× BV elution buffer (50 mm NaH_2_PO_4,_ 300 mm NaCl, 400 mm imidazole, pH 8.0). Elution fractions were pooled and concentrated using an Amicon filter unit with a molecular cutoff of 50 kDa (Merck Millipore, Billerica, MA, United States, #UFC5050). During concentration, buffer exchange to SEC buffer (25 mm HEPES, 150 mm NaCl, pH 6.5) was performed according to the manufacturer's instructions. Further purification of protein samples was performed via SEC.

### Expression and Purification of His‐Tagged GCase‐wt Protein

HEK 293F cells were prepared as described in the previous paragraph and transfected with pCMV‐3‐GBA‐His plasmid. Expression was carried out for a total of 96 h. The conditioned cell supernatant was harvested by centrifugation (1500 × *g*, 10 min, 4 °C) to remove cells and debris, followed by filtration using a 0.22 µm pore size filter unit (Carl Roth, Karlsruhe, Germany, #P666.1). For purification, a single‐use plastic column with filter (ThermoFisher Scientific Inc., Waltham, MA, United States, #29 924) was filled with 1 ml BV of Ni‐NTA beads and equilibrated with equilibration buffer buffer (50 mm NaH_2_PO_4_, 300 mm NaCl, pH 7.4). The harvested cell supernatant was applied to the column via gravity flow at 4 °C. Afterward, the column was washed with 20x BV wash buffer (50 mm NaH_2_PO_4_, 300 mm NaCl, 30 mm imidazole, pH 7.4) by gravity flow. Elution was done with 30× BV elution buffer (50 mm NaH_2_PO_4_, 300 mm NaCl, 200 mm imidazole, pH 7.4). Elution fractions were pooled and concentrated using an Amicon filter unit with a molecular cutoff of 30 kDa (Merck Millipore, Billerica, MA, United States, #UFC5050). During concentration, buffer exchange to storage buffer (25 mm HEPES, 150 mm NaCl, pH 6.0) was performed according to the manufacturer's instructions.

### Expression of GCase Variants in HEK 293F Cells and Concentration of Conditioned Medium for Functional Analyses

For each GCase variant (wt, E326K, N370S, L444P), 20 mL of HEK 293F culture was prepared and transfected with the respective expression construct as described. After 48 h, the culture was harvested via centrifugation and filtration (see previous paragraph). Fifteen milliliters of each culture were then concentrated to <1 mL using an Amicon filter unit with a molecular cutoff of 30 kDa (Merck Millipore, Billerica, MA, United States, #UFC9030). After concentration, PIC was added to prevent sample degradation and all concentrates were adjusted to a volume of 1 mL with Freestyle 293F medium to achieve a final concentration factor of 15× for all samples.

### Expression and Purification of FusionRed‐Tagged L2H5‐wt Protein

Competent One Shot BL21 DE3 pLysS (ThermoFisher Scientific Inc., Waltham, MA, United States, # C606010) were transformed with pT7‐FRed‐L2H5‐wt plasmid via heat shock (42 °C, 45 s) and streaked onto an LB‐agar plate containing ampicillin (100 µg mL^−1^) and chloramphenicol (25 µg µL^−1^). A single colony was picked from to inoculate an overnight culture (37 °C, 125 rpm orbital shaking). The next day, 15 mL of overnight culture was used to inoculate 250 mL of 2YT Medium (Carl Roth, Karlsruhe, Germany, #X966.1) containing ampicillin (100 µg mL^−1^) and chloramphenicol (25 µg µL^−1^). After 60 min of culturing, a final concentration of 1 mm isopropyl *β*‐D‐1‐thiogalactopyranoside (IPTG; AppliChem, Darmstadt, Germany, # A1008) was added for induction. Induction was carried out for 4 h under normal culturing conditions before the bacteria were pelleted (4000 × *g*, 20 min, 4 °C), the medium was discarded and the pellet was suspended in 15 mL of lysis buffer (50 mm NaH_2_PO_4_, 300 mm NaCl, 0.1% Triton X‐100, 1x PIC). Lysis was performed via sonication. The suspension was pulsed five times for 30 s with 30 s of incubation on ice between pulses to avoid heating of the sample. After centrifugation (12 000 × *g*, 20 min, 4 °C), the supernatant was transferred to a 15 mL tube. Ni‐NTA purification of FRed‐L2H5 was done in a batch approach as described in for purification of LIMP‐2/GCase on the previous page. A BV of 500 µL was used. The eluted protein was then concentrated using an Amicon ultra 0.5 filter unit with a 10K cutoff (Merck Millipore, Billerica, MA, United States, #UFC5010) Further purification was performed via SEC.

### Size Exclusion Chromatography (SEC)

For SEC of LIMP‐2/GCase complex and His‐tagged GCase‐wt, a Superdex 200 Increase 3.2/300 column (Cytiva, Marlborough, MA, United States, #28 990 946) was operated on an ÄKTA Pure system (Cytiva Marlborough, MA, United States). The system tubing and flow path were optimized to reduce void volume. Runs were done in SEC buffer at a flow rate of 0.05 mL min^−1^. Twenty‐five microliters of protein solution were injected using a 100 µL loop. Fractions of 100 µL were collected. UV_280_ data was presented as raw mAU values (Figure 3E; Figure S4H, Supporting Information). Based on the UV elution profile, GCase activity assays, and western blot analysis, fractions containing target proteins were selected for further analyses.

SEC of FRed‐L2H5‐wt was performed using a Superdex 200 Increase 10/300 GL column (Cytiva, Marlborough, MA, United States, #28 990 944) in PBS at a flow rate of 0.5 mL min^−1^. Two hundred microliters of protein solution were injected using a 500 µL loop. Fractions of 1000 µL were collected. Based on the UV_280_ and UV_580_ elution profiles, fraction 15 containing monomeric FRed‐L2H5‐wt was concentrated using a 10K Amicon ultra 0.5 filter unit.

### SDS PAGE and Western Blot Analysis

SDS PAGE was performed using a Tris‐glycine buffer system (resolving buffer: ThermoFisher Scientific Inc., Waltham, MA, United States, #HC2215; stacking buffer: ThermoFisher Scientific Inc., Waltham, MA, United States, #HC2115; running buffer: 25 mm Tris, 192 mm glycine, 1% w/v SDS). All samples were prepared by adding 5× Laemmli buffer (0.3 m Tris‐HCl, pH 6.8, 10% w/v SDS, 50% v/v glycerol, 5% v/v *β*‐mercaptoethanol, 5% w/v bromophenol blue) to the samples in a 1:5 ratio and heating the samples for 5 min at 95 °C. The samples were then separated over a 10% polyacrylamide gel. After SDS PAGE, wet blotting was performed to transfer the proteins onto a PVDF membrane (Merck Millipore, Billerica, MA, United States, #IPFL00010). The membrane was blocked in 2% w/v fish gelatin (Merck Millipore, Billerica, MA, United States, #G7041) in Tris‐buffered saline (TBS; 100 mm Tris‐HCl, 685 mm NaCl, pH 7.5) for 1 h at RT. Primary antibodies (see below for dilution) were applied overnight at 4 °C. After incubation, membranes were washed three times with TBS‐T (TBS + 0.1% v/v TWEEN‐20) before incubation with secondary antibodies for 2 h at RT followed by three more washing steps with TBS‐T. The fluorescence‐based acquisition was done using an Odyssey (LI‐COR Biosciences, Lincoln, NE, United States) or IBright (ThermoFisher Scientific Inc., Waltham, MA, United States) imaging system. All antibodies were diluted in TBS + 2% w/v fish gelatin + 0.1% v/v TWEEN‐20. Following antibodies and dilution factors were used for western blot analysis: Primary: goat anti LIMP‐2 (polyclonal, ThermoFisher Scientific Inc., Waltham, MA, United States, #PA5‐19111; dilution: 1:1000), mouse anti GCase (monoclonal, clone E2E, Abnova, Taipeh, Taiwan,#H00002629‐M01; dilution: 1:1000), rabbit anti GCase (polyclonal, Sigma‐Aldrich, St. Louis, MO, United States, #G4171; dilution: 1:1000), rabbit anti LAMP‐2A (monoclonal, clone SA46‐01, Novus Biologicals, Centennial, CO, United States, #NBP2‐67298; dilution: 1:1000), goat anti GAPDH (polyclonal, R&D Systems, Minneapolis, MN, United States, #AF5718; dilution: 1:5000), rabbit anti calnexin (polyclonal, Cell Signaling Technology, Danvers, MA, United States, #2433, dilution: 1:1000). Secondary: donkey anti goat Alexa Fluor 680 (ThermoFisher Scientific Inc., Waltham, MA, United States, #A21084; dilution: 1:10 000), IRDye 800CW donkey anti goat (LI‐COR Biosciences, Lincoln, NE, United States, #926‐32214; dilution: 1:10 000) donkey anti mouse Alexa Fluor 680 (ThermoFisher Scientific Inc., Waltham, MA, United States, #A10038; dilution: 1:10 000), donkey anti rabbit Alexa Fluor 680 (ThermoFisher Scientific Inc., Waltham, MA, United States, #A10043; dilution: 1:10 000).

Quantification of western blot data was performed in ImageStudioLite 5.2 (LI‐COR Biosciences, Lincoln, NE, United States). Intensity data was obtained via a rectangular selection of protein bands and calculation of median signal intensity after background correction. If applicable, lysate samples were normalized to their respective loading control (GAPDH). To correct for differences in intensity values across different image acquisitions, intensity measurements from each replicate were normalized internally to either a reference sample or the overall signal intensity of all samples.

### Staining of Total Protein in Acrylamide Gels

Staining of acrylamide gels after SDS PAGE and western blot was carried out using Coomassie brilliant blue G‐250 (CBB, Carl Roth, Karlsruhe, Germany, #9598.1). The staining was performed following the protocol of Dyballa and Metzger.^[^ ^71^ ^]^ Acquisition of CBB gels was done using the 700 channel of the Odyssey imaging system (see above).

### GCase Enzyme Activity Assays

Activity of GCase‐containing samples (recombinant protein, cell lysate, LE fractions, conditioned culture medium) was measured using the synthetic substrate 4‐methylumbelliferyl‐*β*‐D‐glucopyranoside (4MU, Merck Millipore, Billerica, MA, United States, #M3633). Twenty milliliters of the sample (cell supernatant, SEC fractions) or 2.5 µg of total protein from whole‐cell lysates were added to the well of a black 96‐well Maxisorp plate (ThermoFisher Scientific Inc., Waltham, MA, United States, #437 111). The reaction volume was then increased to 100 µL with GCase activity buffer (see paragraph “Cell Lysis”) and a final concentration of 1 µm of 4MU substrate was added. After incubation at 37 °C for 30 min, the reaction was stopped by adding 100 µL of stop solution (0.1 m glycine, pH 10.4). GCase activity was quantified via fluorescence of released 4‐methylumbelliferone (endpoint measurement) using an Infinite 200 Pro (TECAN, Männedorf, Switzerland), SpectraMax Gemini (Molecular Devices, San José, CA, United States) or ClarioStar (BMG LABTECH, Ortenberg, Germany) multiplate reader (*λ*_ex_: 365 nm; *λ*_em_: 445 nm).

For all activity assays in the presence of recombinant LIMP‐2 or synthetic peptides, sodium taurocholate, and Triton X‐100 were omitted from the reaction to not hinder protein–protein interaction. Figures representing detergent‐free data are Figure 4E,F and Figure S1C (Supporting Information).

For evaluation, all data sets obtained for GCase enzymatic activity were baseline‐corrected and normalized to the overall signal (either total signal or average of controls) within each replicate experiment to correct for signal differences between different measurement devices and replicate measurements.

### Live Cell Measurement of Lysosomal GCase Activity

GCase activity inside the lysosomes of living cells was measured via the fluorescent probe PFB‐Gluc (ThermoFisher Scientific Inc., Waltham, MA, United States, #P11947). The experimental procedure was adapted from Cuddy and Mazzulli.^[^ ^72^ ^]^ Primary human fibroblasts were seeded at 20 000 cells per well on a black 96‐well cell culture dish with transparent bottom (Corning, Corning, NY, United States, #3603). For each replicate, two wells were seeded. After 24 h, the medium of each well was replaced with 100 µL of fresh medium containing the respective treatment (DMSO, 10 µm L2H5‐3xD, 10 µm L2H5‐wt). The next day, the medium was changed again to 50 µL of fresh media containing treatment. On day 3, one of the two‐seeded wells for each replicate (control well) was treated with 200 nm bafilomycin 1A (Baf, Santa Cruz Biotechnology, Dallas, TX, United States, #sc‐201550A) for 1 h to inhibit lysosomal enzyme activity. Then, the medium in all wells was changed once more to 50 µL of fresh medium containing treatment, 200 µg mL^−1^ of PFB‐Gluc and Baf (only in control wells). After 1 h of incubation with the fluorogenic probe, all wells were aspirated, washed 1x with medium, and filled with 100 µL of Fluorobrite DMEM (Gibco, Carlsbad, CA, United States, #A1896701) + 15% v/v FCS containing treatment and bafilomycin (only control wells). Immediately after, the first fluorescence measurement was taken (PFB‐Gluc: *λ*_ex_: 485 nm; *λ*_em_: 538 nm; cutoff: 495 nm). The cells were kept at 37 °C and measured every 30 min for a total of 180 min. Afterward, the cells were washed with PBS and fixed in PBS + 4% w/v formaldehyde (VWR Chemicals, Radnor, PA, United States, #28 794.295) for 20 min at RT. To measure cell volume, the fixed cells were treated with 100 µL of CellTag 700 (LI‐COR Biosciences, Lincoln, NE, United States, # 926‐41090) diluted 1:1000 in blocking buffer (PBS + 0.3% Triton X‐100 + 2% BSA (Sigma‐Aldrich, St. Louis, MO, United States, #A9647) + 5% FCS) for 1 h at RT. Then, all wells were washed 3× with PBS and the plate was scanned in an Odyssey scanner.

For data evaluation, PFB‐Gluc measurements over time were baseline‐corrected and then normalized per well to the respective CellTag 700 signal (cell volume). Replicates were arranged in an XY diagram with their respective Baf control and the AUC for both curves was calculated. Lysosomal activity was then calculated by subtracting the baf control (non‐lysosomal activity) AUC from the non‐inhibited AUC (total activity).

### Immunofluorescence

Primary human fibroblasts were seeded on glass cover slides and cultured until confluent (3–4 days) before fixation in PBS + 4% w/v formaldehyde for 20 min at RT. The cells were permeabilized in ice‐cold methanol for 10 min and washed three times in PBS. Blocking was done in PBS + 5% v/v donkey serum (PANbiotech, Aidenbach, Germany, #P30‐0101) + 0.3% v/v Triton X‐100. Primary antibodies were diluted in antibody buffer (PBS + 1% w/v BSA + 0.3% Triton X‐100) and incubated overnight. After washing three times with PBS, secondary antibodies were diluted in antibody buffer and applied for 1 h at RT. Finally, the slides were washed three more times in PBS before mounting in DAPI Fluoromount‐G (Southern Biotech, Birmingham, AL, United States, #0100‐20). Following antibodies and dilutions were used: primary: goat anti‐LIMP‐2 (polyclonal, ThermoFisher Scientific Inc., Waltham, MA, United States, #PA5‐19111; dilution: 1:100), mouse anti‐GCase (monoclonal, clone E2E, Abnova, Taipeh, Taiwan,#H00002629‐M01; dilution: 1:100); secondary: donkey anti‐goat IgG Alexa Fluor 488 (Invitrogen, Waltham, MA, United States, #A11055, dilution: 1:500), Donkey anti‐Mouse IgG Alexa Fluor 568 (Invitrogen, Waltham, MA, United States, # A10037, dilution: 1:500).

Imaging was done using an LSM 780 confocal laser scanning microscope (Carl Zeiss, Oberkochen, Germany) using a 40× oil objective (Carl Zeiss, Oberkochen, Germany, #420762‐9900‐000). Colocalization in the form of Pearson's correlation coefficient *r* was calculated using the Coloc 2 plugin of ImageJ Fiji.^[^ ^73^ ^]^ Acquired images were segmented by hand into single cells and *r* was determined for each cell. Background signal was determined from a control slide that was not incubated with primary antibodies and the background signal was subtracted from each image before analysis.

### Tracking Lysosomal Delivery of FRed‐L2H5‐wt in Primary Human Fibroblasts

Primary human fibroblasts were cultured on glass cover slides as described above. For labeling of lysosomes, cells were transduced with CellLight Lysosomes‐GFP (Invitrogen, Waltham, MA, United States, # A10037) following the manufacturer's instructions. Twenty‐four after transduction, the cells were treated with 0.25 µg µL^−1^ of recombinant FRed‐L2H5‐wt for 2 h. Afterward, the cells were washed twice in PBS before fixation in PBS + 4% w/v formaldehyde for 20 min at RT. The cover slides were washed three times in PBS before mounting in DAPI Fluoromount‐G. Images were acquired using an LSM 780 confocal laser scanning microscope (Carl Zeiss, Oberkochen, Germany) using a 63× oil objective (Carl Zeiss, Oberkochen, Germany, #420780‐9970‐000).

### Proteome Analysis: Mass Spectrometry of LE Fractions

HEK 293T cells were seeded on 10 cm dishes. When confluence was reached, the cells were treated with 5 µm of L2H5‐wt for 2 and 24 h before harvesting. LE fractions were generated as described before (see “Expression and enrichment of lysosomes from HEK 293T cells”). Proteome analysis was performed as described in the following:

### Enzymatic Protein Digestion

The LE fractions were processed using the single‐pot, solid‐phase‐enhanced sample‐preparation (SP3) approach.^[^ ^74^ ^]^ The proteins were then digested using trypsin overnight at 37 °C. The resultant peptide solution was purified by solid phase extraction in C18 StageTips.^[^ ^75^ ^]^

### Liquid Chromatography Tandem Mass Spectrometry

Samples were analyzed using an Orbitrap Exploris 480 mass spectrometer (Thermo Fisher Scientific, Waltham, MA, United States) coupled to an EASY‐nLC 1200 UHPLC system (Thermo Fisher Scientific, Waltham, MA, United States). Peptides were separated in an in‐house packed 60‐cm analytical column (inner diameter: 75 µm; ReproSil‐Pur 120 C18‐AQ 1.9‐µm silica particles, Dr. Maisch, Ammerbuch, Germany) by online reversed‐phase chromatography through a 120‐min gradient of 2.4–32% v/v acetonitrile with 0.1% v/v formic acid at a nanoflow rate of 250 nl min^−1^. The eluted peptides were sprayed directly by electrospray ionization into the mass spectrometer. Mass spectrometry measurement was conducted in data‐dependent acquisition mode using a top20 method with one full scan (resolution: 60 000, scan range: 300–1650 m/z, target value: 3 × 106, maximum injection time: 28 ms) followed by 20 fragmentation scans via higher energy collision dissociation (HCD; normalized collision energy 30%; resolution: 15 000, target value: 1 × 105, maximum injection time: 28 ms, isolation window: 1.4 m/z). Only precursor ions of +2 to +6 charge state were selected for fragmentation scans. Additionally, precursor ions already isolated for fragmentation were dynamically excluded for 30 s.

### Mass Spectrometry Data Processing

The mass spectrometry raw files were processed in MaxQuant software (version 2.1.3.0).^[^ ^76^ ^]^ Spectra were searched using the Andromeda search engine^[^ ^77^ ^]^ against a target‐decoy database containing the forward and reverse protein sequences of UniProt H. sapiens reference proteome (release 2022_04; 102601 entries), the L2H5‐wt peptide and a default list of common contaminants. Trypsin/P specificity was assigned. Carbamidomethylation of cysteine was set as a fixed modification. Methionine oxidation and protein N‐terminal acetylation were chosen as variable modifications. A maximum of 2 missed cleavages were tolerated. The “second peptides” and “match between runs” options were activated. The minimum peptide length was set to be 7 amino acids. The false discovery rate was set to 1% at both peptide and protein levels.

The MaxLFQ algorithm^[^ ^78^ ^]^ was employed for label‐free protein quantification using its default normalization option. The minimum LFQ ratio count was set to be one. Both the unique and razor peptides were used for quantification. To compare the amounts of L2H5‐wt and LIMP‐2 detected at the peptide level, peptide intensities were log10‐transformed and normalized by median‐centering, assuming the overall amounts of peptides to be similar across all the samples.

### Multiscale Thermophoresis (MST)

For interaction measurements of GCase with LIMP‐2, recombinant GCase (Cerezyme) was labeled with the red fluorescent dye NT‐637 using amine‐reactive protein labeling kit RED‐NHS Monolith (NanoTemper, Munich, Germany, #MO‐L011) following the manufacturer protocol. MST measurements were carried out in 50 mm sodium citrate + 0.05% v/v Tween‐20 (pH: 7.0) with a fixed target (GCase) concentration of 50 nm. For each replicate, a twofold dilution series of 16 samples was measured, starting at 12 µm ligand (recombinant sLIMP‐2‐FC) concentration. Thermophoresis was measured at ambient temperature using a Monolith NT.115 instrument (NanoTemper, Munich, Germany) with both LED and MST power set to 40%. Four replicates were performed for the measurement.

For interaction of GCase with L2H5‐wt peptide, recombinant GCase was produced in‐house (see paragraph “Expression and Purification of His‐tagged GCase‐wt Protein”) and labeled as described for Cerezyme. MST measurements were taken in 50 mm sodium citrate + 0.05% v/v Tween‐20 (pH: 7.4) with a fixed target (GCase) concentration of 25 nm. For each replicate, a twofold dilution series of 16 samples was measured, starting at 125 µm ligand (L2H5‐wt) concentration. Thermophoresis was measured at ambient temperature with LED power set to 40% and MST power set to 60%. Three replicates were performed for the measurement.

The data was analyzed using NT analysis software (NanoTemper, Munich, Germany). Ligand titration resulted in a gradual change in the MST signal, which was plotted as ΔFnorm against the ligand concentration. A binding curve was fitted using a Kd model to derive the binding constant.

### Transformation of Numerical Data

Transformation of numerical data was performed using Excel 2019 (Microsoft, Redmond, WA, United States) numerical data presented in this study mostly represented baseline‐corrected, normalized, or otherwise transformed data. Detailed information was provided in the respective method paragraph.

### Statistical Analyses

Statistical analyses and data presentation were done in GraphPad Prism version 8 (Graphpad Software, Inc., San Diego, CA, United States). Normality of data was assumed and parametric tests were used to determine significance. For the comparison of multiple data groups, regular or nested one‐way or two‐way analysis of variances (ANOVA) was performed with appropriate multiple comparison tests (Dunnett, Tukey, or Sidak). In all cases, the null hypothesis was rejected at *p < 0.05*. For each dataset, the specific statistical analysis is mentioned in the corresponding figure legend.

All graphs presented individual replicates as dots with columns representing mean values ± SEM as error bars unless otherwise specified in the figure legend.

### Modeling of Protein Structures

Molecular illustrations and analyses of protein structures were performed with UCSF Chimera (Resource for Biocomputing, Visualization and Informatics, University of California, San Francisco, with support from NIH P41‐GM103311).^[^ ^79^ ^]^

## Conflict of Interest

The authors declare no conflict of interest.

## Author Contributions

J.P.D., S.B., R.M., L.S., M.R., S.W., S.P., P.A., and F.Z. performed experiments. J.P.D., S.B., D.J., S.W., J.C., M.D., U.H., and F.Z. performed data analysis. J.P.D., P.A., and F.Z. wrote the manuscript. J.P.D., S.B., R.M., D.J., A.M., P.A., and F.Z. contributed to the discussion of data. A.M. provided technical resources. P.A. and F.Z. provided funding. P.A. and F.Z. supervised the study and were responsible for the study design. All authors agreed to the final version of the manuscript.

## Supporting information

Supporting Information

## Data Availability

The data that support the findings of this study are available from the corresponding author upon reasonable request.
