# Supplementary material for: Activation and Purification of ß‐Glucocerebrosidase by Exploiting its Transporter LIMP‐2 – Implications for Novel Treatment Strategies in Gaucher's and Parkinson's Disease
Source: Adv Sci (Weinh). 2024 Apr 26;11(25):2401641. doi: 10.1002/advs.202401641 (PMC11220700; doi:10.1002/advs.202401641)
Supplement: Supplementary file 1 — Supporting Information [file ADVS-11-2401641-s001.pdf]

ADVANCED SCIENCE
Open Access

Supporting Information

for *Adv. Sci.*, DOI 10.1002/advs.202401641

Activation and Purification of ß-Glucocerebrosidase by Exploiting its Transporter LIMP-2 – Implications for Novel Treatment Strategies in Gaucher's and Parkinson's Disease

Jan Philipp Dobert, Simon Bub, Rebecca Mächtel, Dovile Januliene, Lisa Steger, Martin Regensburger, Sibylle Wilfling, Jia-Xuan Chen, Mario Dejung, Sonja Plötz, Ute Hehr, Arne Moeller, Philipp Arnold and Friederike Zunke**

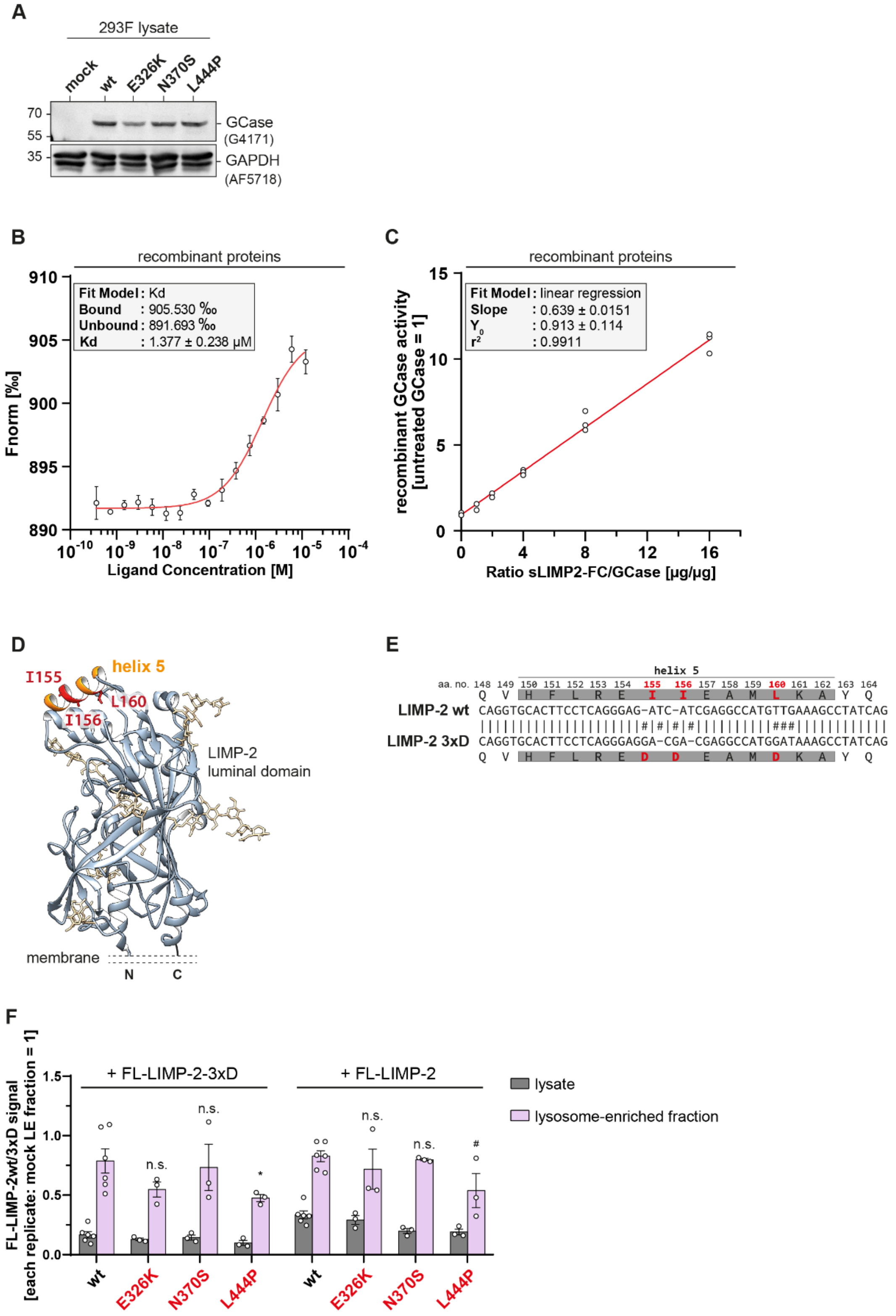

Figure S1: Interaction of GCase and LIMP-2.

A: Expression control western Blot of HEK 293F cells overexpressing GCase variants. All three variants show a similar band at around 65 kDa. **B**: Plot of MST measurements using fluorescently labeled imiglucerase (Cerezyme) and recombinant sLIMP-2-FC (SinoBiological) as a ligand (n = 4).

Measurements were taken at pH = 7.0. A non-linear regression (red line) was fit to the data, from which a Kd value of 1.377 ± 0.238 µM was estimated for the interaction at the given conditions. **C**: Activity of imiglucerase in presence/absence of increasing amounts of recombinant FC-tagged LIMP-2 ectodomain (sLIMP-2-FC) (n = 3). With increasing amounts of sLIMP-2-FC added, GCase activity increased substantially in a linear manner. **D**: Protein structure of the LIMP-2 ectodomain (PDB: 4Q4B) [67]. Helix 5, which mediates GCase interaction is colored orange. Amino acid changes introduced in the 3xD variant are labeled and highlighted in red. Glycosylations are indicated in grey. The dotted line indicates how full-length LIMP-2 would be inserted in the membrane. **E**: Sequence alignment of LIMP-2 wt and LIMP-2-3xD cDNA (excerpt). Amino acid changes are highlighted in red. #: base pair mismatch. **F**: FL-LIMP-2/-3xD levels in HEK 293T cells co-expressing FL-LIMP-2/3xD and GCase variants. Quantification of western blot data shown in Figure 1D. Cells which co-expressed L444P GCase showed reduced levels of both FL-LIMP-2-wt and 3xD variant compared to cells expressing wt GCase. For the E326K and N370S variants, no significant change in FL-LIMP-2 levels could be observed. Asterisks (*) indicate significant differences compared to the wt + FL-LIMP-2-3xD lysosome-enriched fraction. Pound signs (#) indicate significant differences compared to the wt + FL-LIMP-2-wt lysosome-enriched fraction. Statistics: Mean (dot) ± SEM (**B**); replicates (dots) (**C**); replicates (dots) with mean (column) ± SEM (**F**). Tests: Non-linear regression Kd model (**B**); linear regression (**C**), Two-way ANOVA with Dunnett's multiple comparison test (** p<0.05; # p<0.05; n.s.: not significant*).

CTRL-1 [*UKERf1E4-1*]

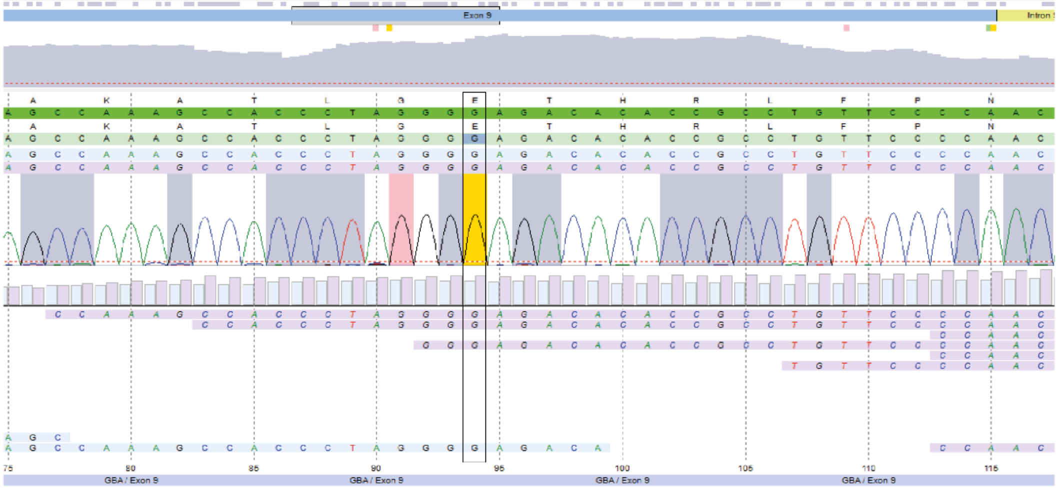

CTRL-2 [*UKERfO3H-1*]

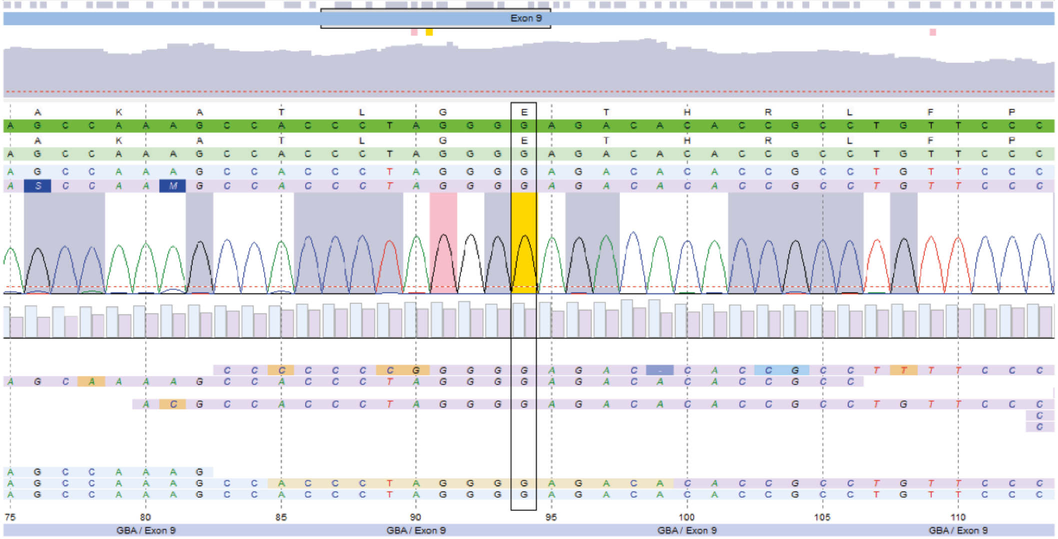

CTRL-3 [*UKERf4CC-1*]

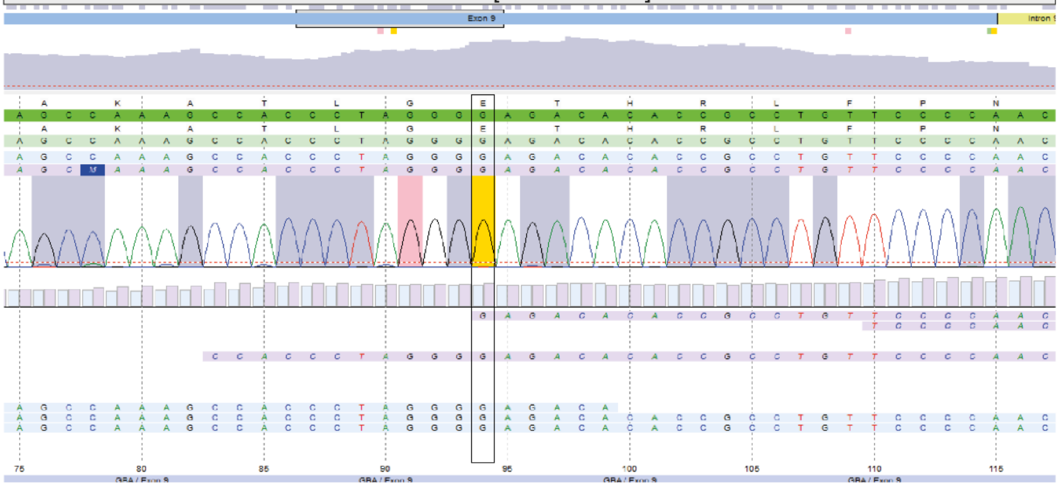

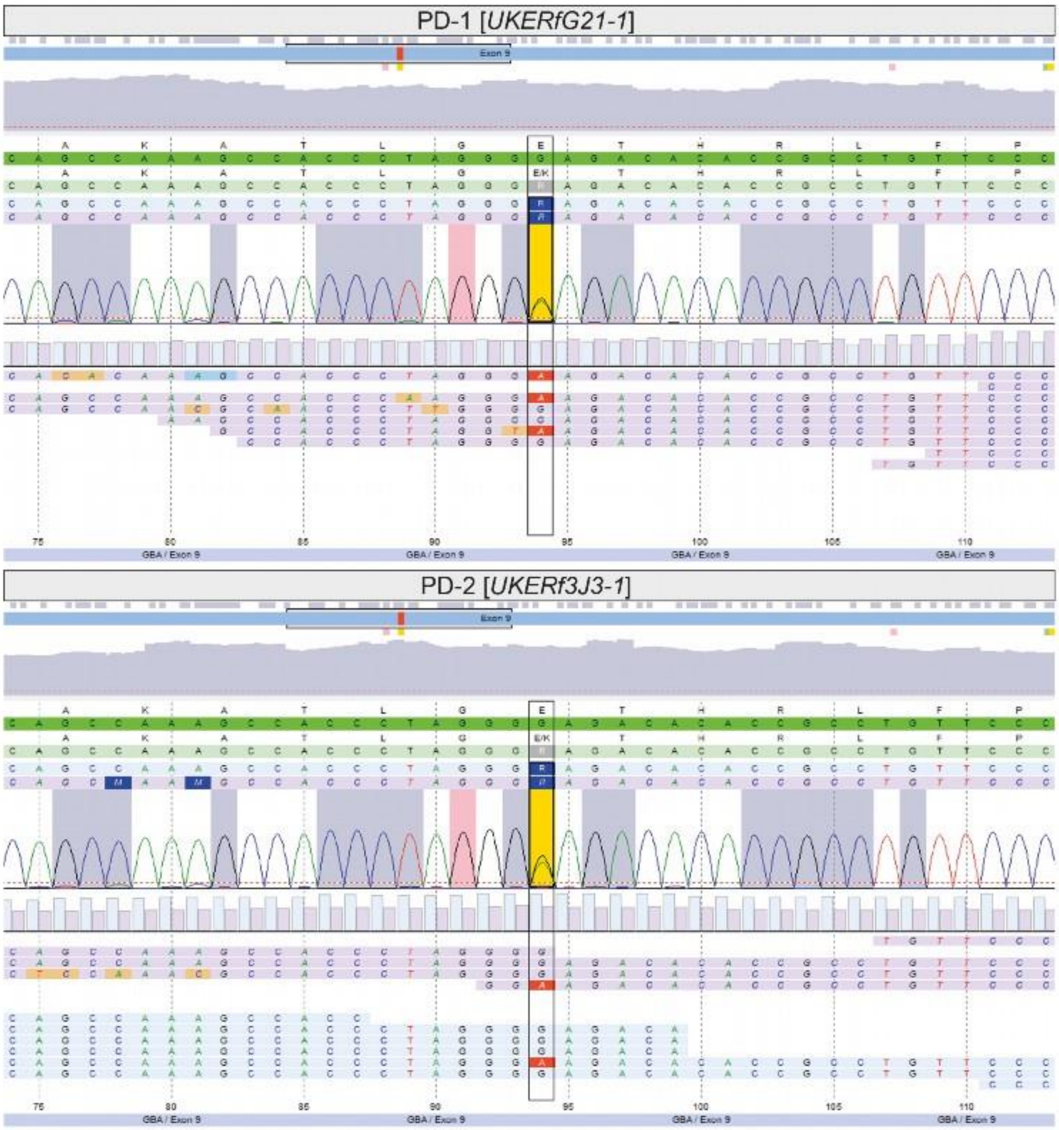

Figure S2: Exome sequencing of primary human fibroblast cell lines.

Snippets of the GBA1 gene in the five fibroblast cell lines CTRL-1 (UKERf1E4-1), CTRL-2 (UKERfO3H-1), CTRL-3 (UKERf4CC-1), PD-1 (UKERfG21-1) and PD-2 (UKERf3J3-1) obtained via whole exome sequencing. The snippets depict a region in exon 9 where SNP rs2230288 (p.E326K) was confirmed on one of the two alleles in the cell lines PD-1 and PD-2, respectively. This SNP was not found in any of the controls and no other SNPs in the GBA1 gene were found for either cell line.

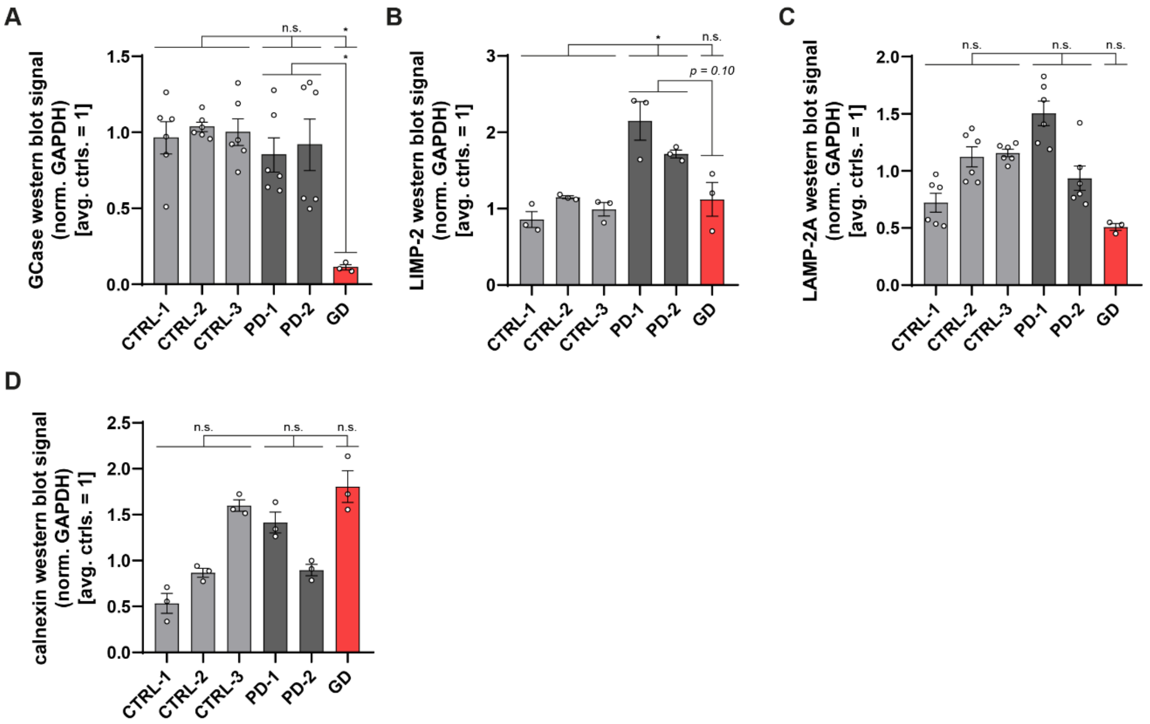

Figure S3: Quantification of protein levels in primary human fibroblasts

A-D: Quantification of GCase (**A**), LIMP-2 (**B**), LAMP-2A (**C**) and calnexin (**D**) in whole-cell lysates of primary human fibroblasts (CTRL, PD and GD). Obtained from western blot data shown in Figure 2A. GCase levels (**A**) across CTRL and PD lines were similar, while the GD line only showed low GCase expression. LIMP-2 levels (**B**) were increased in PD lines compared controls. LAMP-2A (**C**) and calnexin (**D**) levels showed high variance between lines and no significant difference was visible between CTRL, PD and GD groups. Statistics: replicates (dots) with mean (column) ± SEM (**A, B, C, D**). Nested one-way ANOVA (**A, B, C, D**) with Tukey's multiple comparison test (** p<0.05; n.s.: not significant*).

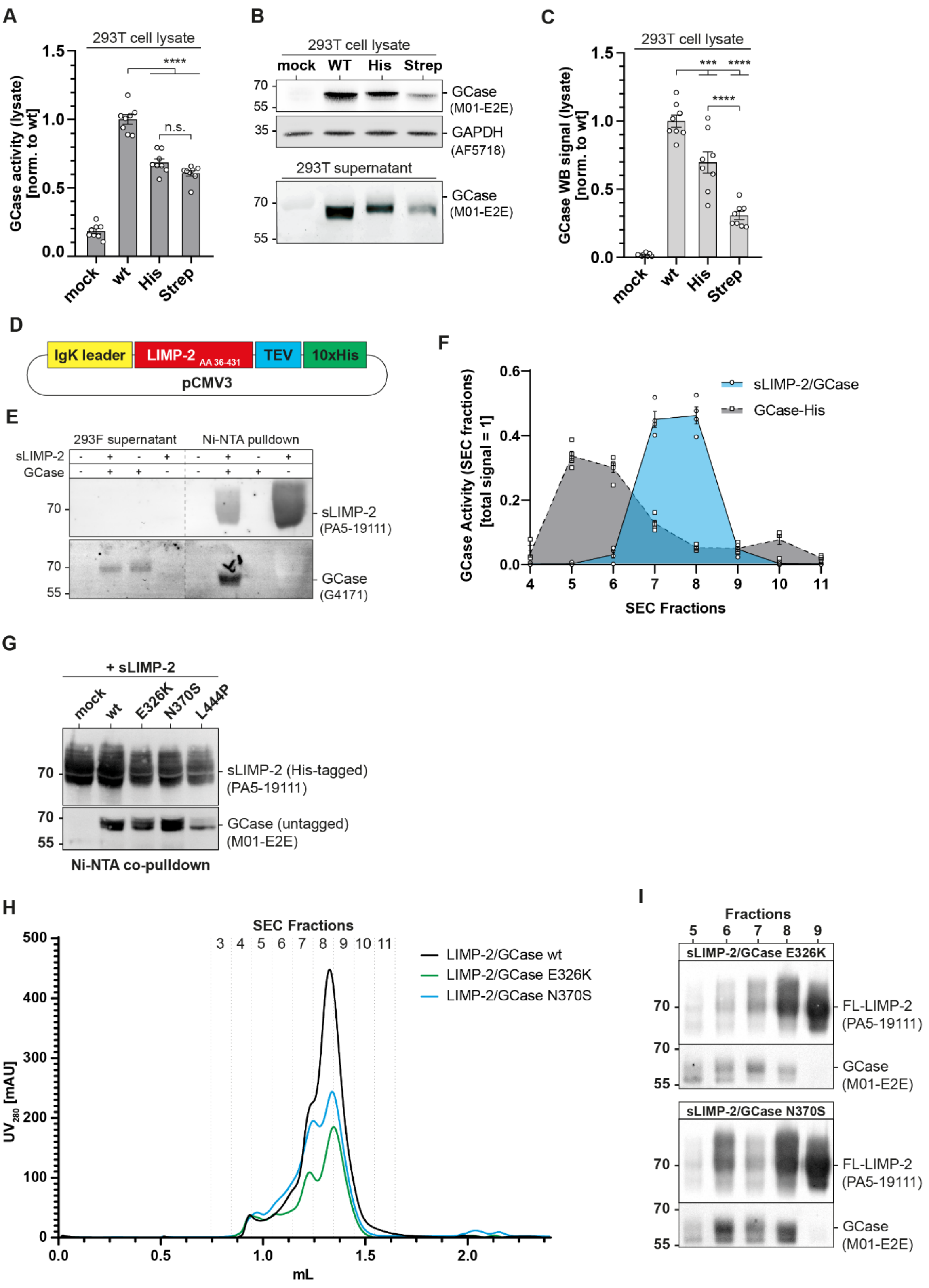

Figure S4: GCase variants and its purification via soluble (s)LIMP-2.

A: GCase Activity in cell lysates of HEK 293T cells overexpressing untagged and tagged GCase constructs (n = 8; individual transfections). Normalized to wt = 1. The activity of tagged GCase variants is significantly reduced compared to the wt. **B**: Representative western blots of cell lysates and supernatants of HEK 293T cells overexpressing different GCase constructs. Tagged GCase variants show

lower band intensity compared to the wt in both lysates and supernatants. Loading controls: GAPDH (lysates), direct blue membrane staining (supernatants). **C**: Quantitative analysis of GCase expression levels in lysates of transfected HEK 293T cells. Expression levels are represented by intensity of GCase band in western blot analysis (n = 8). Normalized to wt = 1. Expression levels of His- and Strep-tagged GCase variants are significantly reduced compared to the wild type **D:** Illustration of sLIMP-2 expression plasmid. The expression cassette comprises the LIMP-2 cDNA for amino acids 36-431 flanked by an N-terminal IgK leader for secretion and a C-terminal TEV cleavage site and 10xHis tag. The vector backbone is pCMV3. **E**: Control western blot of conditioned HEK293F medium and Ni-NTA pulldown after single or co-expression of sLIMP-2 and/or untagged wt GCase. In single expression, His-tagged sLIMP-2 is pulled down while untagged GCase shows no affinity towards the Ni-NTA matrix. In co-expression however, untagged GCase is co-precipitated along sLIMP-2. **F**: GCase activity in SEC fractions of sLIMP-2/GCase sample (n = 3). Activity distribution corresponds to western blot signal with maximum activity in fractions 7/8 for sLIMP-2/GCase and fractions 5/6 for His-tagged GCase. **G**: Western blot showing Ni-NTA co-pulldown of GCase variants along sLIMP-2. N370S was co-precipitated as efficiently as wt GCase, while EE326K levels were slightly reduced and L444P levels heavily reduced compared to the wt. **H**: SEC profiles of sLIMP-2/GCase samples for GCase wt, E326K and N370S. all profiles show the same peaks at fractions 7 and 8/9 corresponding to sLIMP-2/GCase complex and sLIMP-2 monomer respectively. **I**: Western Blot analysis of SEC fractions of sLIMP-2/GCase E326K and N370S. Statistics: replicates (dots) with mean (column) ± SEM (**A, C**); replicates (dots, squares) with mean (line) ± SEM (**F**). Tests: Two-way ANOVA with Tukey's multiple comparison test (**A, C**). **** p<0.001 **** p<0.0001, n.s.: not significant*.

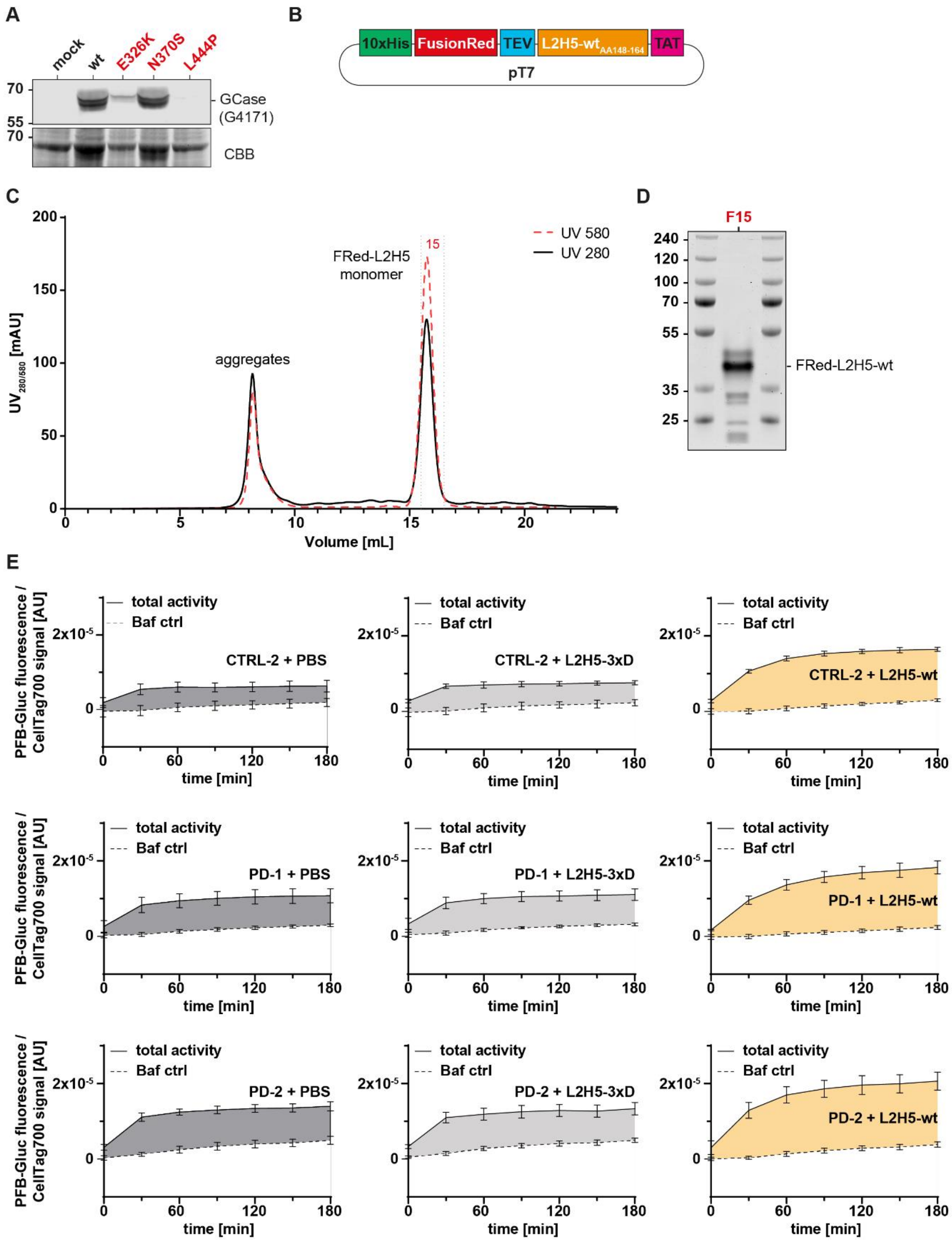

Figure S5: Disease-associated GCase variants and the effect of the LIMP-2-derived peptide in PD and control fibroblasts.

A: Western blot of conditioned HEK293F media (see Figure 4F). Presence of wt, E326K and N370S GCase was confirmed, with levels of E326K reduced compared to wt and N370S. L444P was not detected in the conditioned supernatant, indicating lack of protein stability. **B**: Illustration of pT7-FRed-L2H5-wt plasmid. Amino acids 148-164 of LIMP-2 are fused to FusionRed via a TEV-cleavable linker. The N-terminus harbors a 10xHis tag and the C-terminus is modified with a TAT motif for cell entry. The vector backbone in pT7. **C**: SEC profile of FRed-L2H5 purified from BL21 DE3 pLysS via Ni-NTA

purification. Two elution peaks can be observed in both UV_{280} and UV_{580} spectra, indication presence of FusionRed in both peaks. The earlier peak likely consists of aggregated protein while the second peak represents FRed-L2H5-wt monomer. The fraction collected (fraction 15) is indicated in red. **D**: CBB-stained SDS gel of SEC-purified FRed-L2H5-wt (see Figure S5C for SEC profile). **E**: Comparison of PFB-Gluc turnover over time between fibroblasts lines CTRL-2, PD-1 and PD-2 after control treatment (PBS) and treatment with 10 µM L2H5-3xD or L2H5-wt (n = 3). Each replicate represents one well of cells on a 96-well plate. Substrate turnover is increased in all three cell lines after 48 h of treatment with L2H5-wt. The area between curves (total activity - baf ctrl, summarized in Figure 5D) representing lysosomal GCase activity is indicated in grey (DMSO, L2H5-3xD) or orange (+ L2H5-wt). Statistics: Mean (line) ± SEM (**E**).
